# A Lipid Transfer Protein Increases the Glutathione Content and Enhances *Arabidopsis* Resistance to a Trichothecene Mycotoxin

**DOI:** 10.1371/journal.pone.0130204

**Published:** 2015-06-09

**Authors:** John E. McLaughlin, Mohamed Anwar Bin-Umer, Thomas Widiez, Daniel Finn, Susan McCormick, Nilgun E. Tumer

**Affiliations:** 1 Department of Plant Biology and Pathology, School of Environmental and Biological Sciences, Rutgers University, New Brunswick, New Jersey, United States of America; 2 Bacterial Foodborne Pathogens and Mycology Unit, National Center for Agricultural Utilization Research, United States Department of Agriculture, Agricultural Research Service, Peoria, Illinois, United States of America; Ghent University, BELGIUM

## Abstract

Fusarium head blight (FHB) or scab is one of the most important plant diseases worldwide, affecting wheat, barley and other small grains. Trichothecene mycotoxins such as deoxynivalenol (DON) accumulate in the grain, presenting a food safety risk and health hazard to humans and animals. Despite considerable breeding efforts, highly resistant wheat or barley cultivars are not available. We screened an activation tagged *Arabidopsis thaliana* population for resistance to trichothecin (Tcin), a type B trichothecene in the same class as DON. Here we show that one of the resistant lines identified, trichothecene resistant 1 (*trr1*) contains a T-DNA insertion upstream of two nonspecific lipid transfer protein (nsLTP) genes, *AtLTP4*.*4* and *AtLTP4*.*5*. Expression of both nsLTP genes was induced in *trr1* over 10-fold relative to wild type. Overexpression of *AtLTP4*.*4* provided greater resistance to Tcin than *AtLTP4*.*5* in *Arabidopsis thaliana* and in *Saccharomyces cerevisiae* relative to wild type or vector transformed lines, suggesting a conserved protection mechanism. Tcin treatment increased reactive oxygen species (ROS) production in *Arabidopsis* and ROS stain was associated with the chloroplast, the cell wall and the apoplast. ROS levels were attenuated in *Arabidopsis* and in yeast overexpressing *AtLTP4*.*4* relative to the controls. Exogenous addition of glutathione and other antioxidants enhanced resistance of *Arabidopsis* to Tcin while the addition of buthionine sulfoximine, an inhibitor of glutathione synthesis, increased sensitivity, suggesting that resistance was mediated by glutathione. Total glutathione content was significantly higher in *Arabidopsis* and in yeast overexpressing *AtLTP4*.*4* relative to the controls, highlighting the importance of *AtLTP4*.*4* in maintaining the redox state. These results demonstrate that trichothecenes cause ROS accumulation and overexpression of *AtLTP4*.*4* protects against trichothecene-induced oxidative stress by increasing the glutathione-based antioxidant defense.

## Introduction

Infection of small grain cereals such as wheat and barley with *Fusarium graminearum* or *Fusarium culmorum* causes Fusarium head blight (FHB), also known as scab, which is one of the most important diseases of cereals worldwide. FHB results in yield reductions and in the contamination of grain with heat stable trichothecene mycotoxins, the most prominent being deoxynivalenol (DON) [1–3]. Trichothecenes present a significant food safety risk and health hazard to humans and animals [4], and controlling their accumulation in small grains remains a huge challenge. Strict limits are set for DON levels in flour products and in malting barley and the crop is rejected by the industry if these limits are exceeded. Since the re-emergence of FHB in the USA in 1993, epidemics and associated mycotoxin contamination have been reported in wheat in different production regions [5]. Breeding for resistance to FHB has been challenging due to the potency and persistent nature of the mycotoxins, the need to introgress major resistance loci from exotic germplasm, and the multigenic nature of resistance [1,6]. Major quantitative trait loci (QTLs) like the *Fhb1* region identified on the short arm of chromosome 3B from the wheat cultivar Sumai 3 are rare and the resistance of *Fhb1* is dependent on the genetic background of the cultivar [6,7]. Control of FHB by the application of fungicides has been only partially effective [8].

Trichothecene mycotoxins function as virulence factors during pathogenesis of *F*. *graminearum* on wheat [9,10]. Gene disruption mutants of *F*. *graminearum*, which lack the trichothecene biosynthesis gene, trichodiene synthase (∆*tri5*), are less virulent [9,10]. Hence resistance to trichothecenes is considered to be an important aspect of resistance to FHB. Partial resistance to FHB in wheat is characterized by either inhibition of initial infection (type I) or inhibition of the spread of infection (type II). DON has been shown to promote the spread of *F*. *graminearum* in the rachis of wheat [11]. In barley however, the spread of the infection within the spike is inhibited due to type II resistance [11]. Therefore, strategies that prevent initial infection and inhibit spread of the disease are needed for effective resistance against FHB in wheat and barley.

Trichothecenes target ribosomal protein L3 at the peptidyltransferase center in *S*. *cerevisiae* and inhibit peptidyltransferase activity of eukaryotic ribosomes [12–14]. Expression of a modified ribosomal protein L3 provided enhanced resistance to DON in tobacco and to FHB in wheat [15–17]. Additional studies demonstrated that increasing resistance of wheat to DON enhances resistance to FHB, indicating that genes that confer resistance to DON reduce the impact of FHB [18,19].

Trichothecenes are reported to have diverse roles in the cell that are not limited to the inhibition of cytosolic protein synthesis [20,21]. DON targets the innate immune system and activates ribotoxic stress, resulting in upregulation of cytokine gene expression in mammalian cells [22]. Trichothecene exposure in humans and animals can cause immunosuppression, anorexia, emesis, growth retardation and in large doses, death [20]. In plants DON causes oxidative stress damage by increasing reactive oxygen species (ROS) levels [21]. DON treatment of wheat induced hydrogen peroxide synthesis and caused an increase in programmed cell death [23]. Expression of ROS-related genes such as glutathione-S-transferase, superoxide dismutase, and peroxidase were altered in wheat or barley in response to DON or *F*. *graminearum* [24–27]. Hydrogen peroxide enhances DON production by *Fusarium* [28,29]. Moreover, upon infection with *F*. *graminearum* carbohydrate and nitrogen metabolism of the plant is affected [30]. Plant nitrogen metabolism is redirected towards the formation of polyamines, which induce DON production by *F*. *graminearum* [31].

We previously carried out a genome-wide screen of the *S*. *cerevisiae* deletion collection against trichothecin (Tcin), a type B trichothecene in the same class as DON, and identified mitochondrial translation inhibition and mitochondrial fragmentation as novel mechanisms of trichothecene toxicity [32]. Tcin inhibited translation in isolated mitochondria from yeast, demonstrating that mitochondrial translation is a direct target of trichothecene mycotoxins [33]. We demonstrated a vital role for mitochondrial oxidative stress in trichothecene sensitivity and mitophagy in elimination of trichothecene-damaged mitochondria [34]. Here we screened an activation tagged population of *Arabidopsis thaliana* against trichothecin (Tcin), a type B trichothecene in the same class as DON and identified a gain-of-function mutant in which two closely linked non-specific lipid transfer protein (nsLTP) genes were overexpressed. Overexpression of *AtLTP4*.*4* in *Arabidopsis* or in *S*. *cerevisiae* enhanced resistance to Tcin. Tcin treatment increased reactive oxygen species (ROS) production and caused an imbalance of the redox state in wild type *Arabidopsis* and yeast. Glutathione content was higher and ROS levels were attenuated in both systems when *AtLTP4*.*4* was overexpressed.

## Results

### Identification of trichothecin resistant *Arabidopsis* gain-of-function mutants

**Fig 1:**
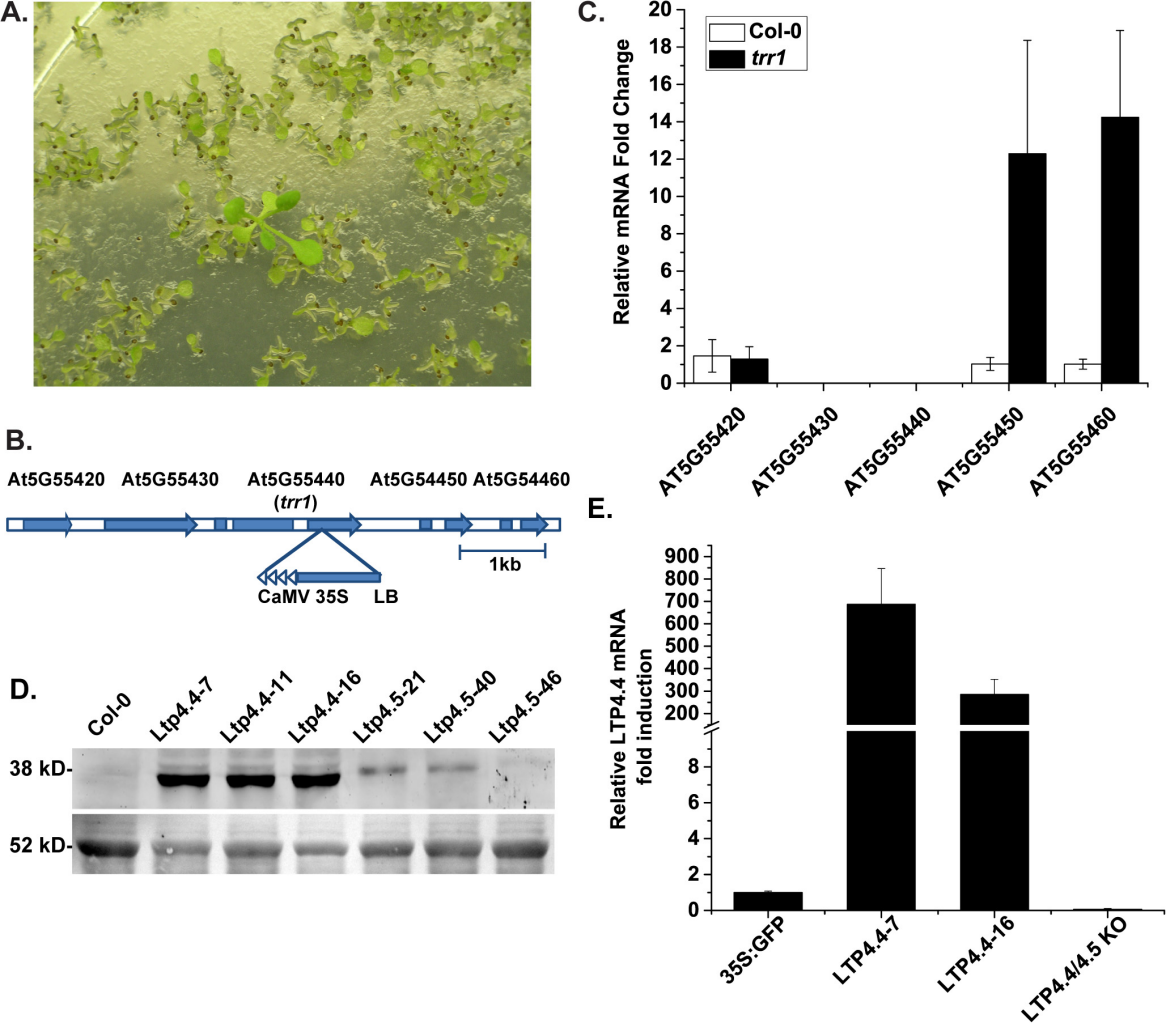
Characterization of *trr1*. (A) Screening of the activation tagged *Arabidopsis* lines identified a mutant, which was able to form roots/leaves on 4 μM Tcin. (B) The T-DNA tag was inserted into the last exon of the At5G55440 gene in *trr1*. (C) Expression analysis of the activation tagged AT5G55440 locus and the flanking genes, At5G55420, At5G55430, At5G55450 (*AtLTP4*.*4*), At5G55460 (*AtLTP4*.*5*) and wild type *Arabidopsis* Col-0 by qRT-PCR. Expression levels of *AtLTP4*.*4* and *AtLTP4*.*5* were significantly higher in the activation tagged *trr1* line compared to the wild type Col-0. (D) Immunoblot analysis of total protein (25 μg) isolated from Col-0 and 3 independent *Arabidopsis* lines containing *AtLTP4*.*4*:*GFP* or *AtLTP4*.*5*:*GFP*, separated on a 12% SDS polyacrylamide gel and probed with monoclonal anti-His IgG (1:500) (GenScript) followed by Amersham ECL Plex C anti-mouse IgG (1:2500) conjugated with Cy3 and scanned using the Typhoon FLA 9500 (GE Healthcare Life Sciences). The blot was stained with Ponceau S and the ~52 kD band is shown for equal loading. (E) Analysis of *AtLTP4*.*4* RNA expression in two independently transformed *Arabidopsis* lines and in the LTP4.4/LTP4.5 knockout line (SALK_207859c).

To investigate the mechanisms underlying trichothecene resistance, we screened activation tagged *Arabidopsis thaliana* (Col-0) seeds for resistance to 4 μM Tcin (1.33 mg/L). This concentration of Tcin was chosen because treatment of *Arabidopsis* (Col-0) leaves with 4 μM Tcin resulted in uniform leaf bleaching in detached leaf assays and caused ~90% inhibition of root growth in germination assays and strong inhibition of vegetative growth. This concentration of Tcin is also similar to the IC_50_ for Tcin in yeast [32] as shown in Table 1. A population of ~250,000 M2 generation *Arabidopsis* seeds transformed with the T-DNA vector, pSKI015, which contains four copies of the cauliflower mosaic virus (CaMV) 35S enhancer [35], were screened. One of the resistant lines identified in our screen, *trr1* (*trichothecene resistant 1*) grew on agar containing 4 μM Tcin, which inhibited germination and root growth of the wild type, Col-0 (Fig 1A). The untreated plants from the resistant line were indistinguishable from the untreated wild type plants. To identify the flanking sequences of the T-DNA insertion locus in *trr1*, thermal asymmetric interlaced (TAIL) PCR was used [35]. Sequence analysis indicated that the T-DNA tag was inserted into the last exon of the At5G55440 gene (Fig 1B), which encodes a protein of unknown function. To determine the effect of the insertion on transcription in this region, expression of At5G55440, two upstream genes (At5G55420 and At5G55430) and two downstream genes (At5G55450 and At5G55460) was analyzed using qRT-PCR with primers specific for each gene (S1 Table). There was little difference in expression of At5G55420 and no expression was detected from At5G55430 or At5G55440 in either *trr1* or in wild type. However in *trr1*, expression of At5G55450, which is ~220 bp downstream of the T-DNA insert was induced 12-fold and expression of At5G55460, which is 424 bp downstream of At5G55450, was induced 14-fold relative to wild type (Fig 1C). At5G55450 and At5G55460 encode two novel non-specific lipid transfer protein (nsLTP) genes, which belong to type IV nsLTPs previously designated as *AtLTP4*.*4* and *AtLTP4*.*5*, respectively [36].

**Table 1:** IC_50_ values for trichothecenes in yeast expressing *Arabidopsis* nsLTPs.

	T-2	DAS	Tcin	DON
Vector Control	127 ± 6 μM	256 ± 5 μM	2.8 ± 0.4 μM	1.3 ± 0.1 mM
*AtLTP4*.*4*	182 ± 15 μM*	353 ± 6 μM***	4.7 ± 0.5 μM*	1.7 ± 0.2 mM*
*AtLTP4*.*5*	154 ± 7 μM*	304 ± 27 μM	3.7 ± 0.7 μM	1.6 ± 0.1 mM
*AtLTP1*.*1*	NA	NA	2.6 ± 0.4 μM	NA

Error indicates S.E where n = 3 independent replicates. Two-sample unpaired t-tests for equal means were performed between the different vectors containing the nsLTPs relative to the vector control for each toxin

*P<0.05

***P < 0.001.

To determine if overexpression of *AtLTP4*.*4* or *AtLTP4*.*5* was responsible for Tcin resistance in *trr1*, C-terminal GFP fusions of each gene were constructed and cloned downstream of the CaMV 35S promoter in the pEarleyGate 103 vector [37]. *Arabidopsis* Col-0 plants were transformed with *AtLTP4*.*4*:*GFP* and *AtLTP4*.*5*:*GFP* using the floral dip method [38]. Homozygous lines for each construct and the vector were identified based on segregation on media containing BASTA. *AtLTP4*.*4*:*GFP* and *AtLTP4*.*5*:*GFP* overexpressing lines were phenotypically normal and indistinguishable from wild type plants. Immunoblot analysis showed that *AtLTP4*.*4*:*GFP* (39.4 kDa) was expressed at a higher level than *AtLTP4*.*5*:*GFP* (40.1 kDa) in three independently generated transgenic lines with each vector (Fig 1D). Analysis of *AtLTP4*.*4* mRNA levels in lines #7 and #16 by qRT-PCR indicated that line #7 showed a 690-fold increase and line #16 showed a 290-fold increase in expression relative to the vector control, 35S:GFP (Col-0) (Fig 1E). The *AtLTP4*.*4/AtLTP4*.*5* knockout line (SALK_207859c), which was phenotypically similar to wild type *Arabidopsis* (Col-0), did not express *AtLTP4*.*4* (Fig 1E).

### *Arabidopsis* overexpressing *AtLTP4*.*4* shows improved resistance to Tcin

**Fig 2:**
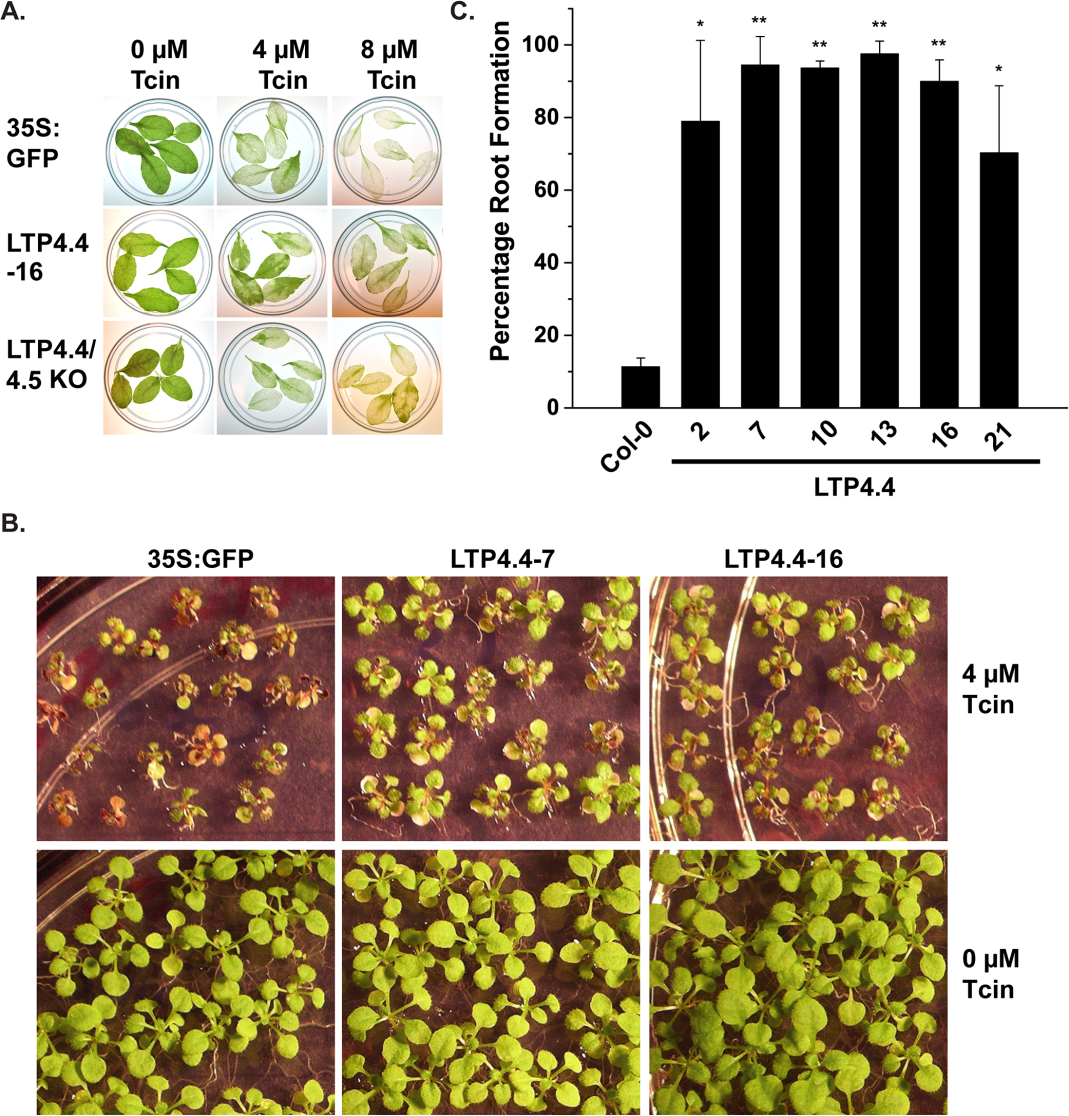
Overexpression of *AtLTP4*.*4* in *Arabidopsis* increases tolerance to Tcin. (A) Detached leaves overexpressing *AtLTP4*.*4* (line #16) relative to 35S:GFP (Col-0) control and the LTP4.4/4.5 KO line after treatment with 4 and 8 μM Tcin for 72 h. (B) Germination of *AtLTP4*.*4* overexpressing lines # 7 and # 16 on 3 μM Tcin relative to the control, 35S:GFP. (C) Percentage of wild type and *AtLTP4*.*4* overexpressing lines able to form roots on 4 μM Tcin in 2 weeks. Error bars represent standard error (S.E.) calculated from three independent experiments. One-way ANOVA with post hoc Bonferroni tests, *P < 0.05, **P<0.01.

Sensitivity of the transgenic lines to Tcin was examined using detached leaf assays in liquid media and by germination assays on solid media. The detached leaf assay showed that the *AtLTP4*.*4* overexpressing line #16 was partially protected from leaf bleaching caused by 4 μM and 8 μM Tcin (2.66 mg/L) relative to the 35S:GFP (Col-0) control (Fig 2A). The *LTP4*.*4/4*.*5* combined knockout line (SALK_207859c) did not appear to impact sensitivity to the toxin compared to the vector control (Fig 2A). When transgenic lines were germinated on solid media containing Tcin, *AtLTP4*.*4* overexpressing transgenic lines #7 and #16 had improved germination on media containing 3 μM Tcin (~1.0 mg/L) compared to 35S:GFP (Col-0) control (Fig 2B). *AtLTP4*.*5* overexpressing lines were not as resistant as the *AtLTP4*.*4* overexpressing lines and were not analyzed further. The percentage of *AtLTP4*.*4* overexpressing lines able to form roots two weeks after exposure to 4 μM Tcin, determined by counting ~100 seedlings per line, and is shown in Fig 2C. Six different transgenic lines overexpressing *AtLTP4*.*4* displayed significantly greater root growth at 14 days after plating compared to the control. These results demonstrate that *AtLTP4*.*4* overexpressing lines show improved resistance to Tcin.

### Localization of AtLTP4.4 and AtLTP4.5 proteins in *Arabidopsis*

**Fig 3:**
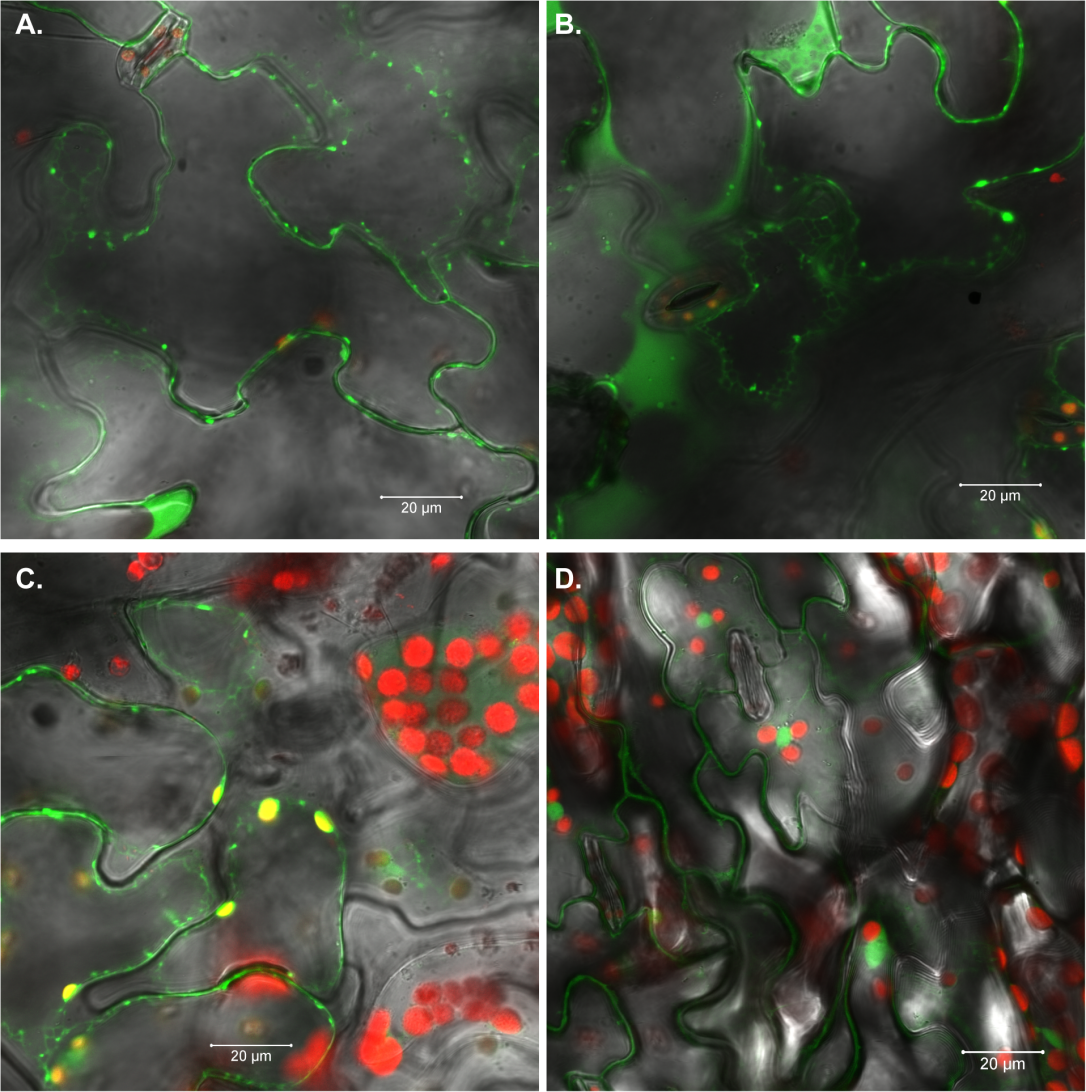
AtLTP4.4:GFP is localized to the cell wall/apoplast and chloroplasts in *Arabidopsis* leaves. (A) Confocal microscopy analysis of line #16 expressing AtLTP4.4:GFP showing expression in the cell wall/apoplast, (B) possibly in the ER, and (C) chloroplasts (D) 35S:GFP (Col-0) control.

Analysis of transgenic *Arabidopsis* leaves expressing *AtLTP4*.*4*:*GFP* (line #16) by confocal microscopy revealed that the GFP signal accumulated in the cell wall and in the apoplast (Fig 3A) and expression was also detected in the roots (S1 Fig). Intracellular expression of *AtLTP4*.*4*:*GFP* was observed in a network, characteristic of the ER (Fig 3B) and *AtLTP4*.*4*:*GFP* colocalized with the chloroplasts as indicated by the yellow signal (Fig 3C). *Arabidopsis* Col-0 transformed with the 35S:GFP vector revealed no staining around the chloroplast or the ER (Fig 3D). *Agrobacterium*-mediated transient expression analysis of *AtLTP4*.*4*:*GFP* and *AtLTP4*.*5*:*GFP* in tobacco leaves confirmed the subcellular localization observed in the transgenic plants. Transient expression analysis 2 days after *Agrobacterium* infiltration showed that AtLTP4.4:GFP and AtLTP4.5:GFP were localized in the extracellular space near the cell wall (S2 Fig). AtLTP4.4:GFP showed stronger GFP signal than AtLTP4.5:GFP and colocalization of the AtLTP4.4:GFP protein and the chloroplasts was evident by the yellow signal (S2B Fig).

### Overexpression of *AtLTP4*.*4* reduces Tcin-induced reactive oxygen species (ROS) accumulation in *Arabidopsis*

**Fig 4:**
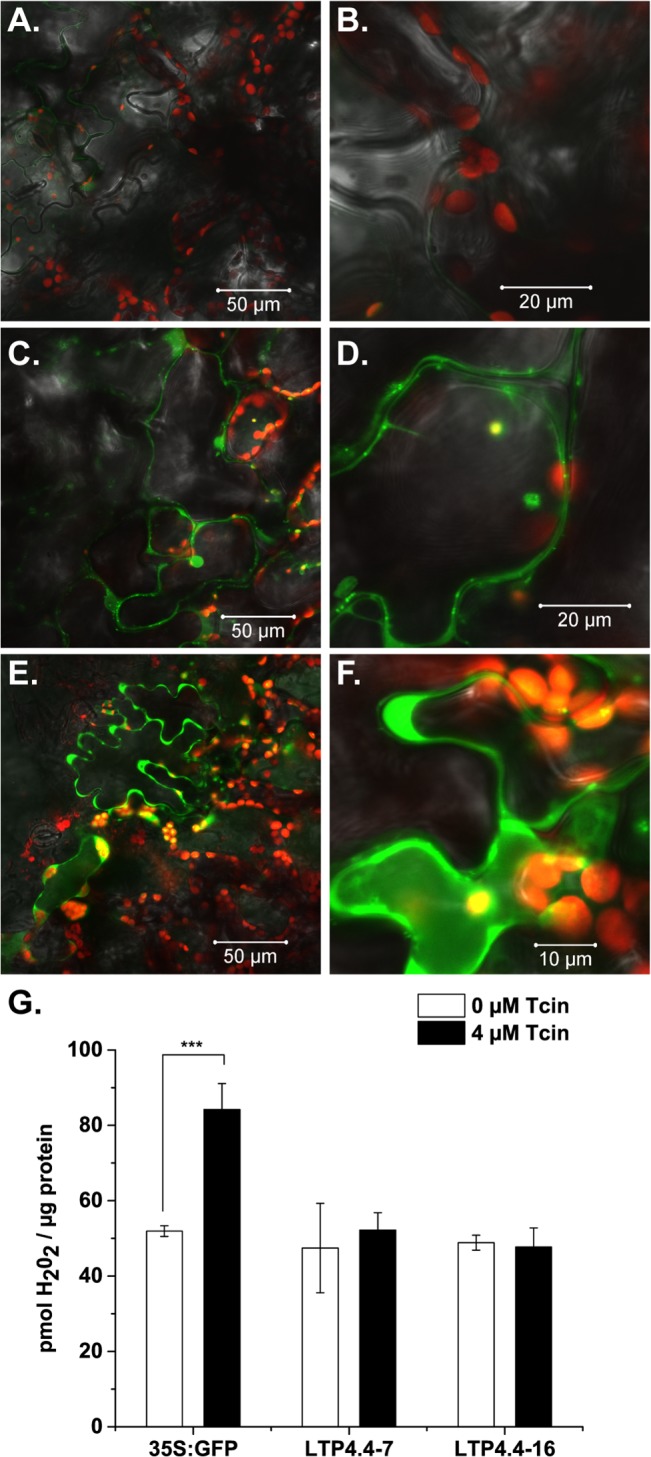
Overexpression of *AtLTP4*.*4* reduces Tcin-induced ROS accumulation in Arabidopsis. Confocal microscopy analysis of ROS accumulation in wild type *Arabidopsis* (Col-0) leaves infiltrated with Tcin and the cell-permeable fluorogenic probe 2'-7'-Dichlorodihydrofluorescein diacetate (DCFH-DA) after 24 h. (A,B) Mock treatment with buffer, (C,D) treatment with 8 μM Tcin (E,F) treatment with 24 μM Tcin. (G) Intact leaf assay to quantify H_2_O_2_ levels in transgenic *Arabidopsis* lines #7 and #16 overexpressing *AtLTP4*.*4* using Amplex Red. Treatment with 4 μM Tcin for 24 h induced H_2_O_2_ accumulation in the vector control (35S:GFP), but not in lines #16 and #7. Error bars indicate S.E where n = 3 independent replicates. One-way ANOVA with post hoc Bonferroni tests, ***P<0.001.

We previously showed that trichothecenes induce mitochondrial ROS accumulation in yeast and exogenous addition of antioxidants alleviate growth inhibition by trichothecenes, indicating that toxicity of trichothecenes is at least partially a consequence of increased ROS production [34]. To determine if Tcin treatment induces ROS in *Arabidopsis*, wild type *Arabidopsis* (Col-0) leaves infiltrated with Tcin for 24 h and then were stained with 2’,7’-dichlorofluorescin diacetate (H2DCF-DA), a non-fluorescent, cell permeable dye, which is retained intracellularly following cleavage by esterases and is converted to the highly fluorescent dichlorofluorescein (DCF) when oxidized by ROS. Confocal microscopy analysis of the mock treatment (Fig 4A and 4B) revealed very light staining without highlighting any particular structure. The 8 μM Tcin treatment showed that DCF stain colocalized with chloroplasts, the cell wall, and possibly the apoplast (Fig 4C and 4D). Increasing the dosage of Tcin to 24 μM (~8 mg/L) intensified the DCF staining of chloroplasts (Fig 4E and 4F) relative to the mock treatment. Similar results were obtained when tobacco leaves were treated with DON. The mock treatment revealed very light staining without highlighting any particular structure in tobacco (S3A Fig). The 24 hour treatment of tobacco leaves with 10 μM DON (2.96 mg/L) revealed colocalization of DCF fluorescence with chloroplasts (S3B Fig). Increasing the concentration of DON to 240 μM intensified the staining of chloroplasts (S3C and S3D Fig). Disorganized chlorophyll autofluorescence was detected in leaf tissues treated with 240 μM DON (71 mg/L) for 24h (S3E Fig). Similarly, 2h treatment with 600 nM paraquat (methyl viologen) (154 μg/L) led to oxidative damage of chloroplast membranes and ultimately to bursting of the chloroplast (S3 Fig).

We investigated if treatment of wild type *Arabidopsis* (Col-0) leaves with antioxidants would protect against Tcin, *Arabidopsis* leaves were treated with 8 μM Tcin in the presence of 2 mM vitamin C, vitamin E, or para-amino benzoic acid (PABA). Treatment with antioxidants partially protected against the bleaching effects of Tcin (S4A Fig). Incubation of *Arabidopsis* Col-0 seedlings with liquid MS media containing Tcin revealed photobleaching after 16 h light/8 h dark treatment (S4B Fig). In contrast, continuous dark treatment provided protection from photobleaching caused by Tcin (S4B Fig). These results showed that Tcin treatment induces ROS accumulation in *Arabidopsis* and provided evidence that ROS accumulation and light play a role in Tcin-mediated tissue damage.

To determine if Tcin treatment leads to ROS accumulation in transgenic *Arabidopsis* overexpressing *AtLTP4*.*4*, intact leaves from 35S:GFP control plants and from *AtLTP4*.*4* overexpressing lines #7 and #16 were exposed to 0 (mock) and 4 μM Tcin, incubated for 24 h and H_2_O_2_ levels were quantified with Amplex Red Ultra (Molecular Probes). A significant increase in H_2_O_2_ was observed when the 35S:GFP control line was treated with 4 μM Tcin relative to the mock treatment (Fig 4G). In contrast, H_2_O_2_ level did not increase when *AtLTP4*.*4* overexpressing lines were treated with 4 μM Tcin. The H_2_O_2_ level in lines #7 and #16 was significantly lower relative to the control line (35S:GFP) after exposure to 4 μM Tcin. These results indicate that overexpression of *AtLTP4*.*4* attenuates Tcin-induced ROS accumulation.

### Expression of *AtLTP4*.*4* reduces Tcin induced ROS accumulation and confers resistance to Tcin in yeast

To determine if nsLTP overexpression provided resistance to trichothecenes in *Saccharomyces cerevisiae*, yeast cells overexpressing *AtLTP4*.*4* or *AtLTP4*.*5* were grown on increasing concentrations of type A (T-2 and DAS) or type B (Tcin and DON) trichothecenes and average IC_50_ values were determined (Table 1). The IC_50_ of yeast expressing *AtLTP4*.*4* was significantly higher relative to cells carrying the empty vector (VC) when grown on T-2 toxin, DAS, Tcin or DON, while yeast expressing *AtLTP4*.*5* showed significant resistance to only T-2 toxin (Table 1). To determine if the ability to confer Tcin resistance is specific to *AtLTP4*.*4* and *AtLTP4*.*5*, we investigated whether At2g15050 (*AtLTP1*.*1*), which belongs to type I group [36], mediates resistance to Tcin. The IC_50_ of yeast expressing *AtLTP1*.*1* for Tcin was similar to the vector control, indicating that *AtLTP1*.*1* did not protect yeast against Tcin (Table 1).

**Fig 5:**
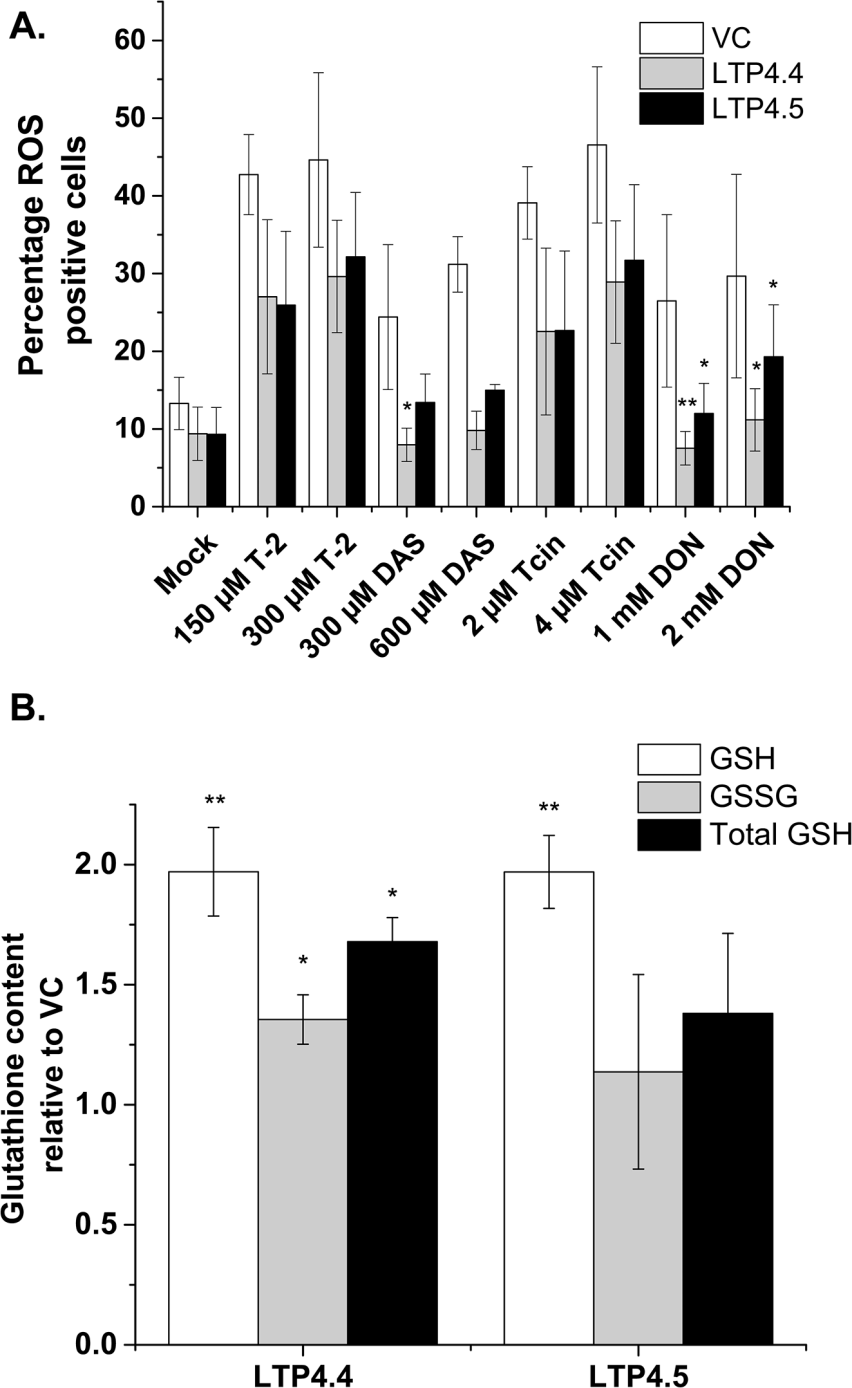
*AtLTP4*.*4* expression attenuates ROS levels and increases glutathione content in yeast. **(** *A*) Trichothecene treated or untreated wild type yeast (BY4743) cells carrying the empty vector, overexpressing *AtLTP4*.*4*, or overexpressing *AtLTP4*.*5* were stained for ROS using DCHF and analyzed by flow cytometry. ANOVA with post hoc Bonferroni tests, *P < 0.05, **P < 0.01. (*B*) GSH and GSSG levels were quantified relative to vector control and normalized to total protein in yeast expressing *AtLTP4*.*4* and *AtLTP4*.*5* after 3 hour galactose induction. ANOVA with post hoc Bonferroni tests, *P < 0.05, **P < 0.01.

We previously showed that treatment with type A (T-2 and DAS) and type B (Tcin and DON) trichothecenes caused a dose-dependent increase in ROS production in yeast [33]. These findings prompted us to explore ROS generation in nsLTP expressing yeast treated with trichothecenes. ROS levels were measured using the ROS-sensitive stain DCFH-DA, which only fluoresces upon contact with ROS. While 13% of the untreated vector control cells stained positive for ROS, this increased to 43–45% with T-2, 24–32% with DAS, 39–47% with Tcin and 26–30% with DON treatment (Fig 5A). Basal ROS levels were lower in *AtLTP4*.*4* and *AtLTP4*.*5* overexpressing cells relative to the vector control across the eight different trichothecene treatments (Fig 5A). ROS positive cells dropped to 26–32% upon T-2 treatment, 8–15% upon DAS treatment, 23–32% upon Tcin treatment and 8–19% upon DON treatment in yeast overexpressing *Arabidopsis* nsLTPs (Fig 5A). These results demonstrate that ROS generation triggered by trichothecenes is attenuated when *Arabidopsis* nsLTPs are expressed in yeast.

### Glutathione levels are higher in *Arabidopsis* and yeast overexpressing *AtLTP4*.*4*.

**Fig 6:**
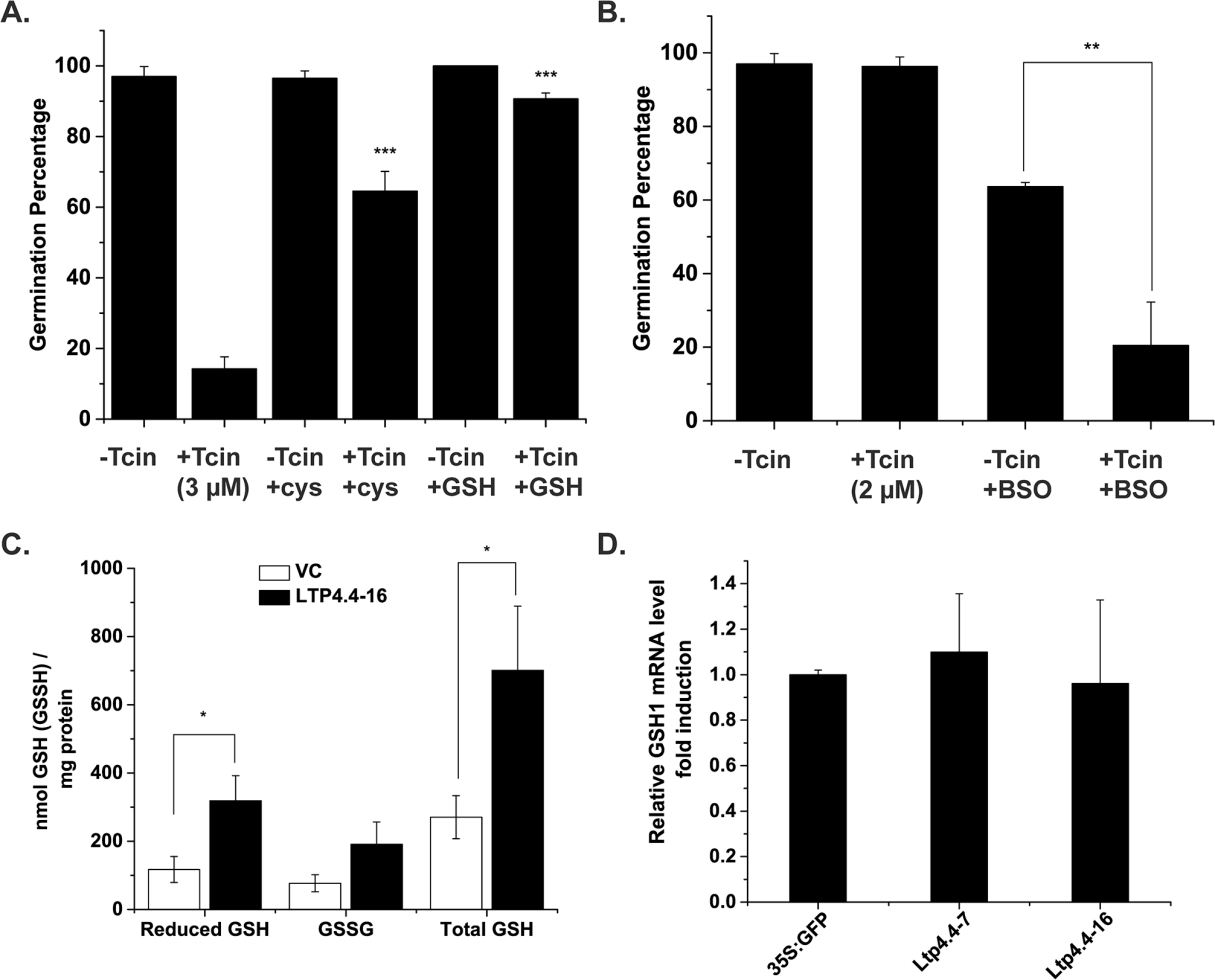
*AtLTP4*.*4* overexpression increases glutathione content of *Arabidopsis* leaves. (*A*) Germination of 35S:GFP seedlings using media supplemented with 1 mM cysteine (Cys) or 250 μM glutathione (GSH) and 3 μM Tcin. Comparison of the effect of the addition of cys and GSH with Tcin to the Tcin treatment alone using ANOVA with post hoc Bonferroni tests, ***P<0.001. (*B*) Germination assay of 35S:GFP seedlings on media supplemented with 250 μM buthionine sulfoximine (BSO) and 2 μM Tcin. Unlike 3 μM Tcin which substantially reduces the germination percentage, 2 μM Tcin did not impact germination percentage. Comparison of the effect of BSO treatment on Tcin treated or untreated samples using ANOVA with post hoc Bonferroni tests, **P < 0.01. (*C*) GSH and GSSG levels of mature leaves from *AtLTP4*.*4* overexpressing line #16 and 35S:GFP control (3 biological replicates each). ANOVA with post hoc Bonferroni tests, *P < 0.05. *(D) GSH1* expression in lines overexpressing *AtLTP4*.*4* relative to 35S:GFP line by qRT-PCR.

Glutathione is a major antioxidant within cells and is known to play a key role in the maintenance of the redox status and the protection against oxidative stress by detoxifying H_2_O_2_ [39]. As shown in Fig 6A, addition of 1 mM cysteine or 250 μM GSH did not have an effect on germination percentage of 35S:GFP control line in the absence of 3 μM Tcin, but significantly enhanced germination percentage of 35S:GFP line in the presence of the toxin relative to 35S:GFP line treated with Tcin alone. Addition of 1 mM cysteine, which is a component of GSH, has been reported to increase the GSH content of *Arabidopsis* by ~3.6-fold [40]. To determine if GSH is an essential mediator of resistance to Tcin, we explored the relationship between GSH levels and trichothecene sensitivity. Germination of wild type *Arabidopsis* (Col-0) was examined on plates containing buthionine sulfoximine (BSO), a potent inhibitor of GSH synthesis, which causes GSH depletion [41]. BSO treatment significantly enhanced the sensitivity of wild type *Arabidopsis* to a low dose (2 μM) of Tcin, which did not affect sensitivity in the absence of BSO (Fig 6B). These results suggested that preventing GSH depletion would lead to protection against Tcin.

We examined the effect of *AtLTP4*.*4* overexpression on the glutathione level in transgenic plants by measuring the GSH and GSSG content of leaves from line #16 using the GSH-Glo Glutathione Assay kit (Promega). Overexpression of *AtLTP4*.*4* resulted in a significant (2.9-fold) increase in reduced glutathione (GSH) and a 2.1-increase in glutathione disulfide (GSSG) in line #16 compared to the vector control (Fig 6C). Total GSH content was significantly higher (2.6-fold) in line #16 relative to the vector control (Fig 6C). To determine if GSH biosynthesis is increased in transgenic plants, we examined the expression of *GSH1*, which encodes glutamate-cysteine ligase, an enzyme that catalyzes the first and rate-limiting step of glutathione biosynthesis in the chloroplast. *GSH1* expression was not significantly higher in transgenic lines #7 and #16 overexpressing *AtLTP4*.*4* (Fig 6D), suggesting that the increase in GSH level was not due to the overexpression of *GSH1*.

To determine if overexpression of *Arabidopsis* nsLTPs affected the GSH level in yeast, the GSH and GSSG content of yeast cells expressing *AtLTP4*.*4* and *AtLTP4*.*5* was analyzed using the GSH-Glo Glutathione Assay kit (Promega). Reduced GSH level was significantly higher in yeast expressing *AtLTP4*.*4* and *AtLTP4*.*5* (Fig 5B). However, yeast expressing only *AtLTP4*.*4* showed a significant increase in the GSSG level and in the total GSH level relative to yeast harboring the vector, but not yeast expressing *AtLTP4*.*5* (Fig 5B). These results demonstrated that the increase observed in the total glutathione content in the *AtLTP4*.*4* overexpressing *Arabidopsis* line translated to yeast.

## Discussion

Activation tagging has not previously been used to isolate genes that can confer resistance to mycotoxins. In this study we screened activation-tagged *Arabidopsis* lines transformed with a T-DNA vector containing four copies of the CaMV 35S enhancer sequences and identified a mutant, *trr1*, which displayed enhanced resistance to Tcin. Analysis of *trr1* revealed that the T-DNA, which contained tandem (4X) CaMV 35S enhancers upregulated expression of two tightly linked downstream genes *AtLTP4*.*4* (AT5G55450) and *AtLTP4*.*5* (AT5G55460) over 10-fold (Fig 1C). The two genes, which encode previously uncharacterized nsLTPs, show 48% nucleotide sequence identity and are arranged in tandem duplication repeats on chromosome 5. *Arabidopsis* contains 49 nsLTP genes, which are classified into nine types based on phylogenetic analysis [36]. *AtLTP4*.*4* and *AtLTP4*.*5* belong to type IV nsLTPs, which include 5 members on chromosome 5 [36]. Although other nsLTP genes were located near *AtLTP4*.*4* and *AtLTP4*.*5*, only *AtLTP4*.*4* and *AtLTP4*.*5* were overexpressed in *trr1*, allowing functional characterization of these two genes without interference from other family members.

The nsLTPs are small basic proteins characterized by a conserved eight cysteine motif backbone [42]. The Cys residues form 4 disulfide bonds that stabilize a hydrophobic cavity, which can bind fatty acid chains and potentially other small molecules [42]. The physiological function of nsLTPs is not well understood. The nsLTPs have been shown to exchange lipids between membranes and artificial vesicles *in vitro* [43]. There is evidence that they are involved in diverse biological processes, such as cutin biosynthesis, anther development, stress resistance and different signaling pathways [43], suggesting that they may be involved in intracellular lipid trafficking. They are generally secreted and associated with the cell wall [42]. We show here that AtLTP4.4:GFP localized not only to the cell wall and apoplast, but also colocalized with the chloroplast and possibly with the ER (Fig 3), providing evidence for intracellular localization of AtLTP4.4:GFP.

Although nsLTPs have antimicrobial activity and are thought to be important components of defense against bacterial and fungal pathogens, their mechanism of action is not well understood. Pepper nsLTPs show structural similarity to elicitins from fungal pathogens [44]. Overexpression of these proteins was shown to activate systemic acquired resistance (SAR)-mediated signaling pathways [45]. The *DIR1* gene of *Arabidopsis*, which encodes another type IV nsLTP (*AtLTP 4*.*1*) [36], was identified in a screen of mutants deficient in SAR, but not in local defense responses [46]. *DIR1* overexpression had little or no effect on resistance to bacterial infection in *Arabidopsis* [47]. Although *DIR1* belongs to the same group as *AtLTP4*.*4* and *AtLTP4*.*5*, we did not see improved resistance to Tcin in the *DIR1* overexpressing *Arabidopsis* line, DD5E [47]. Moreover, overexpression of another type IV nsLTP from *Arabidopsis*, *AtLTP1*.*1* failed to produce resistance to Tcin in yeast (Table 1). These results indicate that there are functional differences between different members of the nsLTP gene family and that *AtLTP4*.*4* and *AtLTP4*.*5* have a function specific to the trichothecene response. *AtLTP4*.*4* was expressed at a higher level in transgenic *Arabidopsis* than *AtLTP4*.*5* and *Arabidopsis* plants overexpressing *AtLTP4*.*4* showed a higher level of resistance. Yeast expressing *AtLTP4*.*4* also showed a higher level of resistance to different trichothecenes compared to yeast expressing *AtLTP4*.*5*. The observed resistance to type A (T-2 and DAS) and type B (Tcin and DON) trichothecenes associated with *AtLTP4*.*4* expression in yeast indicated that resistance was not specific to plants, suggesting a conserved cellular protection mechanism.

Previous results indicated that impairment of mitochondrial function due to trichothecene treatment causes an increase in ROS in yeast [33]. Mitochondria not only accumulate ROS, they are also a target of Tcin-induced ROS because Tcin induced mitochondrial ROS damages mitochondrial membranes in yeast [33]. Several studies suggest that chloroplasts are a major source of ROS in plants [48,49]. Confocal microscopy analysis of *Arabidopsis* and tobacco leaves treated with trichothecenes showed that ROS stain is associated with the chloroplast, the cell wall, and the apoplast after treatment with DON or Tcin. Increasing the dose of Tcin or DON intensified the staining of chloroplasts. Overnight treatment with a high dose of DON (240 μM) damaged chloroplasts as does the treatment with a low concentration of paraquat (S3E and S3F Fig), suggesting that the chloroplast plays an important role in trichothecene sensitivity both as a target of the toxin and as a source of ROS generation. The damage from Tcin appeared to be light-dependent (S4B Fig) similar to Paraquat, which is toxic only in plants exposed to light [50]. These results suggest that Tcin induces photooxidative stress by either directly targeting the chloroplast or by promoting damage to the chloroplast. Application of DON to barley detached leaf segments induced bleaching in plants exposed to light [51]. As observed in our study, bleaching did not occur in samples exposed to dark. However, experimental evidence suggested that damage to the plasmalemma was the primary effect of the DON treatment. Hence, the authors concluded that the light-induced damage to the chloroplast represented a secondary effect [51]. Further evidence for the effect of light on plant responses to trichothecenes has been provided using *Arabidopsis* cell cultures and wheat plants exposed to DON [52]. This study showed that light plays a role in viability and induction of defense responses due to DON exposure, but did not address the direct effect of DON on the chloroplast [52]. Evidence that mycotoxins can damage chloroplasts was observed with fumonisin B1, which functions in a light-dependent manner to induce ROS production in chloroplasts, chloroplast damage, and ultimately cell death in *Arabidopsis* [53].

The protective effect of overexpressing the cysteine-rich *AtLTP4*.*4* could be due to the reduction of membrane damage mediated by trichothecene-induced ROS accumulation. Glutathione is the primary defense compound against oxidative damage to membranes [39]. It reduces hydroperoxide groups on phospholipids and other lipid peroxides through glutathione-S-transferases [39]. We found that overexpression of *AtLTP4*.*4* resulted in increased glutathione levels, suggesting the involvement of *AtLTP4*.*4* in the regulation of cellular redox homeostasis. Exogenous addition of glutathione and other antioxidants enhanced resistance to Tcin while the addition of buthionine sulfoximine (BSO), an inhibitor of glutathione synthesis, increased sensitivity to the toxin, suggesting that Tcin resistance is mediated by glutathione. A significant increase in the total glutathione level was observed in *Arabidopsis* and yeast overexpressing *AtLTP4*.*4*, and both systems showed improved resistance to Tcin. In contrast, a similar increase in the total glutathione level was not observed in yeast overexpressing *AtLTP4*.*5*, consistent with the lower level of trichothecene resistance compared to yeast expressing *AtLTP4*.*4*.

Due to its reducing power, glutathione has essential functions in plant development, defense response against oxidative stress, including redox-homeostatic buffering and detoxification of heavy metals and xenobiotics [54]. Detoxification mechanisms against DON include conjugation to glucose and glutathione [24,55,56]. Detoxifying trichothecenes by overexpressing UDP-glucosyltransferase, an enyzme that converts DON to the less toxic DON-3-O-glucoside, has been shown to provide enhanced resistance in *Arabidopsis*, barley, and wheat [18,57]. Glutathione conjugates of DON have been identified in wheat, indicating that this is an important detoxification mechanism [24,55,56]. The thiol group of glutathione can react with the α,β-unsaturated ketone of type B trichothecenes and form a Michael adduct [58]. The trichothecene conjugates are known as masked mycotoxins since they are undetectable by conventional analytical techniques because their structure has been altered in the plant [59]. They may present a health risk because conversion back to a toxic form can occur upon consumption by animals or humans [59]. However, the glutathione conjugates cannot cross biological membranes and are not likely to revert back to the toxic form [59]. Hence during infection by *F*. *graminearum*, glutathione may be sequestered by conjugation to DON, affecting the oxidative status and the defense response of the host plant.

We show here that enhancing glutathione content impacts plant resistance to trichothecenes and may offer a way to compliment other trichothecene detoxification methods. Emerging evidence suggests that redox sensitive Cys residues in proteins may function as oxidant sensors and can scavenge ROS [60]. Maspin (a serine protease inhibitor) which is rich in cysteine residues (8 in total, similar to nsLTPs) was found to function as an efficient ROS scavenger in mouse mammary cells [61]. Recently an *Arabidopsis* screen using paraquat identified an oxidative stress-induced bioreactive peptide, OSIP108, containing a ROS-scavenging cysteine [62]. The Cys groups in *AtLTP4*.*4* may participate in maintaining the redox state by becoming oxidized by ROS. Our results demonstrate the general ability of *AtLTP4*.*4* to impact the redox system of the cell and protect against trichothecene-mediated oxidative stress by maintaining redox homeostasis.

## Materials and Methods

### Screening activation tagged lines and characterization of the Tcin resistant mutant

The activation tagged *Arabidopsis thaliana* lines in Columbia (Col-0) background were generated by *Agrobacterium* transformation with the pSKI015 plasmid containing the modified T-DNA vector, which contains four copies of the cauliflower mosaic virus (CaMV) 35S enhancer [35], and were a kind gift from Dr. Brian Gregory. *In vitro* germination assays on solid media in petri plates (150 mM) were used to screen for mutants resistant to the toxin. The plates contained 1X Murashige and Skoog (Gamborg Modified) Basal Media (2.5 g/L) (Sigma M0404), 50 mM Ferric EDTA, and 0.6% Phytagel (Sigma P8169) and 4 μM Tcin. Resistance was scored 30 days after seeding the plates. Resistant mutants were planted in MetroMix 360 potting soil (Sun Gro Horticulture), and grown to maturity in a growth chamber maintained at 22°C (16 h light/8 hour dark). The *trr1* was characterized by thermal asymmetric interlaced (TAIL) PCR [63]. The amplified band was sequenced and the genomic location of the insert was mapped using T-DNA express (http://signal.salk.edu/cgi-bin/tdnaexpress).

### Plant expression vectors

The *AtLTP4*.*4* and *AtLTP4*.*5* coding DNA sequence (CDS) without the stop codon were amplified by RT-PCR and cloned into the entry vector pCR8/GW/TOPO (Invitrogen) to generate NT1618 and NT1619, respectively. *AtLTP4*.*4* and *AtLTP4*.*5* without the stop codon were cloned into the binary vector pEarleyGate 103 (BAR-35S-Gateway-GFP-His tag-OCS 3')[37] to generate NT1620 and NT1621, respectively. *Agrobacterium* (GV3101 MP90 strain) containing NT1620 and NT1621 was used to transform *Arabidopsis* [38]. Transformed seedlings were identified by plating out on MS agar media containing BASTA (25 μg/mL).

### Trichothecene mycotoxin

Tcin, DAS, T-2, and DON were isolated and prepared as described previously [32,64].

### Real-time PCR

Quantitative Reverse Transcription PCR (qRT-PCR) using SYBR Green was used to quantify expression of genes in *trr1*. *AtLTP4*.*4* expression was determined in the vector control, overexpression lines, and a knockout line (SALK_207859c, Col-0), which was obtained from the *Arabidopsis* Biological Resource Center (ABRC). Glutamate-cysteine ligase (GSH1, AT4G23100) expression was measured from mature leaf tissue collected from the vector control and overexpression lines. The Comparative Ct method (∆∆Ct) method was used to quantity gene expression using Actin8 for normalization relative to the Col-0 control.

### Transient expression and confocal microscopy analysis

Fluorescence of the lower epidermis of tobacco leaf discs was visualized with a Zeiss LSM 710 Laser Scanning Microscope (Carl Zeiss, Inc., Thornwood, NY, USA) two to three days after infiltration with AtLTP4.4:GFP and AtLTP4.5:GFP. GFP and chlorophyll were excited with a 488-nm argon laser and fluorescence was detected using 495–570 nm and 650–760 nm bandpass filters, respectively. Image data were analyzed using the ZEN 2010 software (Carl Zeiss, Inc.). Leaves from stably transformed *Arabidopsis* lines were visualized with a Zeiss LSM 710 laser scanning microscope as described above for the transient expression analysis in tobacco.

### Yeast Strains and Plasmids

Yeast strain BY4743 (MATa/α, his3Δ1/his3Δ1, leu2Δ0/leu2Δ0, LYS2/lys2Δ0, met15Δ0/MET15, ura3Δ0/ura3Δ0) was used as the background for all experiments. Gateway entry vector pDONR221 was purchased from Invitrogen while the destination vector pAG425-GAL1-ccdB-HA (plasmid# 14249) was purchased from Addgene (www.addgene.com). Standard gateway cloning procedure was followed to clone AtLTP4.4 and AtLTP 4.5 genes into the destination vector, pAG425-GAL1-ccdB-HA to generate NT1754 and NT1755, respectively.

### Measurement of ROS

The quantification of H_2_O_2_ in leaf extracts was performed using Amplex Red Ultra (Invitrogen-Molecular Probes) according to the manufacturer’s instructions. Fluorescence was measured with the BioTek Synergy 4 plate reader (excitation 530 nm, emission 590 nm). The concentration of H_2_O_2_ in each sample was calculated using a standard curve. The standard curve was linear to at least 10 μM H_2_O_2_.

ROS was visualized in leaf tissue by infiltrating the cell permeable fluorescent probe 2′-7′-Dichlorodihydrofluorescein diacetate (DCFH-DA) using vacuum infiltration. Leaf disks were visualized with a Zeiss LSM 710 laser scanning microscope (Carl Zeiss, Inc., Thornwood, NY, USA) as described above.

Treated and untreated yeast cells were stained with 2',7'-dichlorfluorescein-diacetate (DCFH-DA) for ROS generation. Cells were analyzed using the Accuri C6 Flow Cytometer (Accuri Cytometers Inc., Ann Arbor, MI) as described previously [34].

### GSH and GSSG quantification

Reduced glutathione (GSH) and oxidized glutathione disulfide (GSSG) levels were determined using the GSH-Glo Glutathione Assay (Promega) according to the manufacturer’s instructions. Briefly, leaf tissue was quick frozen in liquid nitrogen and ground in an eppendorf tube in 250 μL of PBS (pH 7.4) containing 2 mM EDTA. The protein content of the lysate was determined by the Bradford assay. The lysate was diluted to ~0.05 mg/mL and a 15 μL aliquot was added to two adjacent wells in a 384-well white plate. To one well 1 mM TCEP was added to determine total (GSH and GSSG) glutathione concentration. 15 μl of GSH-Glo Reagent (Luciferin-NT and Glutathione S-Transferase added at a 1/100 ratio to GSH-Glo Reaction Buffer) was added to each sample and incubated at room temperature in the dark for 30 minutes. 15 μl reconstituted Luciferin detection reagent was then added and the sample was incubated for 15 minutes. Luminescence was determined using the BioTek Synergy 4 plate reader and glutathione levels were established by comparing data to a standard curve.

For GSH/GSSH quantification for yeast, cultures were spun down, supernatant removed, quick frozen in liquid nitrogen and stored at -80°C. The yeast pellet was resuspended in 250 μL of PBS (pH 7.4) containing 2 mM EDTA and the cells were broken using glass beads with the MiniBeadBeater (Biospec Products). The protein content of the lysate was determined via UV absorbance using the BioTek Synergy4 spectrophotometer with the Take3 plate. The lysate was diluted to ~0.05 mg/mL concentration of protein and analyzed as described for the plant samples.

### Statistics

The data are shown as means ± SEM and analyzed, where indicated, either by performing t-tests or by ANOVA using the Bonferroni algorithm to test differences between individual treatments. Origin 2015 (OriginLab) software was used for both graphing and for the statistical analysis.

## Supporting Information

S1 Fig *AtLTP4*.*4*:*GFP* expression in the roots of transgenic Arabidopsis plants. Confocal microscopy analysis of root cuttings from wild type *Arabidopsis* Col-0 (A) and *Arabidopsis* line #16 overexpressing *AtLTP4*.*4*:*GFP* (B).
(TIF)

S2 Fig Transient expression of *AtLTP4*.*4*:*GFP* and *AtLTP4*.*5*:*GFP* in tobacco leaves. (A) Mock treated tobacco leaf with autofluorescent (red) chloroplasts. (B) Tobacco leaf infiltrated with *Agrobacterium* containing *AtLTP4*.*4*:*GFP*. (C) Tobacco leaf infiltrated with *Agrobacterium* containing *AtLTP4*.*5*:*GFP*.
(TIF)

S3 Fig DON induces ROS accumulation in wild type tobacco plants. (A) Mock infiltration of tobacco leaves with buffer. (B) Infiltration of tobacco leaves with 10 μM DON for 24h, (C, D and E) infiltration of tobacco leaves with 240 μM DON for 24h. Chloroplast membrane damage is shown in (E) after 240 μM DON treatment for 24h resulting in disorganized chlorophyll autofluorescence. (F) Treatment with 600 nM paraquat for 2h. Colocalization of the DCF fluorescence (green) with chloroplasts (red) is indicated by yellow.
(TIF)

S4 Fig Exogenous antioxidants enhance the tolerance of *Arabidopsis* (Col-0) to Tcin. (A) Wild type *Arabidopsis* (Col-0) leaves were treated with 2 mM vitamin C, vitamin E, or para-amino benzoic acid (PABA) either alone or together with 8 μM Tcin for 48h and photographed. (B) Wild type *Arabidopsis* (Col-0) seedlings were treated with 4 μM or 40 μM Tcin and grown in 16h light/8h dark cycle or in continuous dark cycle for 6 days and photographed.
(TIF)

S1 Table Primers used for qRT-PCR analysis of *trr1* and wild type *Arabidopsis* (Col-0). Primer pair sequences used to amplify At5G55440 and upstream and downstream flanking genes and the housekeeping gene Act8 are shown.
(TIF)

## References

[1] Foroud NA, Eudes F. Trichothecenes in cereal grains. Int J Mol Sci. 2009; 10: 147–173. 19333439 10.3390/ijms10010147 PMC2662451

[2] McMullen M, Jones R, Gallenberg D. Scab of wheat and barley: A re-emerging disease of devastating impact. Plant Dis. 1997; 81: 1340–1348. 10.1094/PDIS.1997.81.12.1340 30861784

[3] Desjardins AE, Hohn TM, McCormick SP. Trichothecene biosynthesis in *Fusarium* species—Chemistry, genetics, and significance. Microbiol Rev. 1993; 57: 595–604. 8246841 10.1128/mr.57.3.595-604.1993 PMC372927

[4] McCormick SP, Alexander NJ, Proctor RH. Trichothecene triangle: toxins, genes, and plant disease Phytochemicals, Plant Growth, and the Environment; 2013 Springer pp. 1–17.

[5] McMullen M, Bergstrom G, DeWolf E, Dill-Macky R, Hersman D, Shaner G, et al A unified effort to fight an enemy of wheat and barley: *Fusarium* Head Blight. Plant Dis. 2012; 96: 1712–1728. 30727259 10.1094/PDIS-03-12-0291-FE

[6] Buerstmayr H, Ban T, Anderson JA. QTL mapping and marker-assisted selection for *Fusarium* head blight resistance in wheat: a review. Plant Breed. 2009;128: 1–26.

[7] Kolb FL, Bai G-H, Muehlbauer GJ, Anderson JA, Smith KP, Fedak G. Host plant resistance genes for *Fusarium* head blight. Crop Sci. 2001; 41: 611–619.

[8] Wegulo SN, Bockus WW, Nopsa JH, De Wolf ED, Eskridge KM, Peiris KH, et al Effects of integrating cultivar resistance and fungicide application on *Fusarium* head blight and deoxynivalenol in winter wheat. Plant Dis. 2011; 95: 554–560. 30731943 10.1094/PDIS-07-10-0495

[9] Proctor RH, Hohn TM, McCormick SP. Reduced virulence of *Gibberella zeae* caused by disruption of a trichothecene toxin biosynthetic gene. Mol Plant Microbe Interact. 1995; 8: 593–601. 8589414 10.1094/mpmi-8-0593

[10] Desjardins AE, Proctor RH, Bai G, McCormick SP, Shaner G, Buechley G, et al Reduced virulence of trichothecene-nonproducing mutants of *Gibberella zeae* in wheat field tests. Molecular Plant Microbe Interact. 1996; 9: 775–781.

[11] Jansen C, von Wettstein D, Schafer W, Kogel KH, Felk A, Maier FJ. Infection patterns in barley and wheat spikes inoculated with wild-type and trichodiene synthase gene disrupted *Fusarium graminearum* . Proc Natl Acad Sci USA. 2005; 102: 16892–16897. 16263921 10.1073/pnas.0508467102 PMC1283850

[12] Fried HM, Warner JR. Cloning of yeast gene for trichodermin resistance and ribosomal protein L3. Proc Natl Acad Sci USA. 1981; 78: 238–242. 7017711 10.1073/pnas.78.1.238 PMC319027

[13] Wickner RB, Ridley SP, Fried HM, Ball SG. Ribosomal protein L3 is involved in replication or maintenance of the killer double-stranded RNA genome of *Saccharomyces cerevisiae* . Proc Natl Acad Sci USA. 1982; 79: 4706–4708. 6750608 10.1073/pnas.79.15.4706 PMC346745

[14] Cundliffe E, Cannon M, Davies J. Mechanism of inhibition of eukaryotic protein synthesis by trichothecene fungal toxins. Proc Natl Acad Sci USA. 1974; 71: 30–34. 4521056 10.1073/pnas.71.1.30 PMC387925

[15] Di R, Blechl A, Dill-Macky R, Tortora A, Tumer NE. Expression of a truncated from of yeast ribosomal protein L3 in transgenic wheat improves resistance to *Fusarium* head blight. Plant Sci. 2010; 178: 374–380.

[16] Harris LJ, Gleddie SC. A modified Rpl3 gene from rice confers tolerance of the *Fusarium graminearum* mycotoxin deoxynivalenol to transgenic tobacco. Mol Plant Pathol. 2001; 58: 173–181.

[17] Di R, Tumer NE. Expression of a truncated form of ribosomal protein L3 confers resistance to pokeweed antiviral protein and the *Fusarium* mycotoxin deoxynivalenol. Mol Plant Microbe Interact. 2005;18: 762–770. 16134888 10.1094/MPMI-18-0762

[18] Shin S, Torres-Acosta JA, Heinen SJ, McCormick S, Lemmens M, Paris MP, et al Transgenic *Arabidopsis thaliana* expressing a barley UDP-glucosyltransferase exhibit resistance to the mycotoxin deoxynivalenol. J Exp Bot. 2012; 63: 4731–4740. 10.1093/jxb/ers141 22922639 PMC3428005

[19] Lemmens M, Scholz U, Berthiller F, Dall'Asta C, Koutnik A, Schuhmacher R, et al The ability to detoxify the mycotoxin deoxynivalenol colocalizes with a major quantitative trait locus for *Fusarium* head blight resistance in wheat. Mol Plant Microbe Interact. 2005; 18: 1318–1324. 16478051 10.1094/MPMI-18-1318

[20] Wu F, Groopman JD, Pestka JJ. Public health impacts of foodborne mycotoxins. Annu Rev Food Sci Technol. 2014;5: 351–372. 10.1146/annurev-food-030713-092431 24422587

[21] Arunachalam C, Doohan FM. Trichothecene toxicity in eukaryotes: cellular and molecular mechanisms in plants and animals. Toxicol Lett. 2013; 217: 149–158. 10.1016/j.toxlet.2012.12.003 23274714

[22] Pestka JJ. Deoxynivalenol-induced proinflammatory gene expression: mechanisms and pathological sequelae. Toxins (Basel). 2010; 2: 1300–1317. 10.3390/toxins2061300 22069639 PMC3153246

[23] Desmond OJ, Manners JM, Stephens AE, Maclean DJ, Schenk PM, Gardiner DM, et al The *Fusarium* mycotoxin deoxynivalenol elicits hydrogen peroxide production, programmed cell death and defence responses in wheat. Mol Plant Pathol. 2008; 9: 435–445. 10.1111/j.1364-3703.2008.00475.x 18705859 PMC6640518

[24] Gardiner SA, Boddu J, Berthiller F, Hametner C, Stupar RM, Adam G, et al Transcriptome analysis of the barley-deoxynivalenol interaction: evidence for a role of glutathione in deoxynivalenol detoxification. Mol Plant Microbe Interact. 2010; 23: 962–976. 10.1094/MPMI-23-7-0962 20521958

[25] Schweiger W, Steiner B, Ametz C, Siegwart G, Wiesenberger G, Berthiller F, et al Transcriptomic characterization of two major *Fusarium* resistance quantitative trait loci (QTLs), *Fhb1* and *Qfhs*.*ifa-5A*, identifies novel candidate genes. Mol Plant Pathol. 2013; 14: 772–785. 10.1111/mpp.12048 23738863 PMC3902993

[26] Walter S, Doohan F. Transcript profiling of the phytotoxic response of wheat to the *Fusarium* mycotoxin deoxynivalenol. Mycotoxin Res. 2011; 27: 221–230. 10.1007/s12550-011-0099-2 23605803

[27] Ding L, Xu H, Yi H, Yang L, Kong Z, Zhang L, et al Resistance to hemi-biotrophic *F*. *graminearum* infection is associated with coordinated and ordered expression of diverse defense signaling pathways. PLoS One. 2011; 6(4): e19008 10.1371/journal.pone.0019008 21533105 PMC3080397

[28] Audenaert K, Callewaert E, Hofte M, De Saeger S, Haesaert G. Hydrogen peroxide induced by the fungicide prothioconazole triggers deoxynivalenol (DON) production by *Fusarium graminearum* . BMC Microbiol. 2010; 10: 112 10.1186/1471-2180-10-112 20398299 PMC2859870

[29] Ponts N, Couedelo L, Pinson-Gadais L, Verdal-Bonnin MN, Barreau C, Richard-Forget F. *Fusarium* response to oxidative stress by H_2_O_2_ is trichothecene chemotype-dependent. FEMS Microbiol Lett. 2009; 293: 255–262. 10.1111/j.1574-6968.2009.01521.x 19239497

[30] Audenaert K, Vanheule A, Hofte M, Haesaert G. Deoxynivalenol: a major player in the multifaceted response of *Fusarium* to its environment. Toxins (Basel). 2014; 6: 1–19. 10.3390/toxins6010001 PMC3920246 24451843

[31] Gardiner DM, Kazan K, Praud S, Torney FJ, Rusu A, Manners JM. Early activation of wheat polyamine biosynthesis during *Fusarium* head blight implicates putrescine as an inducer of trichothecene mycotoxin production. BMC Plant Biol. 2010; 10: 289 10.1186/1471-2229-10-289 21192794 PMC3022911

[32] McLaughlin JE, Bin-Umer MA, Tortora A, Mendez N, McCormick S, Tumer NE. A genome-wide screen in *Saccharomyces cerevisiae* reveals a critical role for the mitochondria in the toxicity of a trichothecene mycotoxin. Proc Nat Acad Sci USA. 2009;106: 21883–21888. 10.1073/pnas.0909777106 20007368 PMC2799815

[33] Bin-Umer MA, McLaughlin JE, Basu D, McCormick S, Tumer NE. Trichothecene mycotoxins inhibit mitochondrial translation—implication for the mechanism of toxicity. Toxins (Basel). 2011; 3: 1484–1501. 10.3390/toxins3121484 22295173 PMC3268453

[34] Bin-Umer MA, McLaughlin JE, Butterly MS, McCormick S, Tumer NE. Elimination of damaged mitochondria through mitophagy reduces mitochondrial oxidative stress and increases tolerance to trichothecenes. Proc Natl Acad Sci USA. 2014; 111: 11798–11803. 10.1073/pnas.1403145111 25071194 PMC4136610

[35] Weigel D, Ahn JH, Blazquez MA, Borevitz JO, Christensen SK, et al Activation tagging in *Arabidopsis* . Plant Physiol. 2000; 122: 1003–1013. 10759496 10.1104/pp.122.4.1003 PMC1539247

[36] Boutrot F, Chantret N, Gautier MF. Genome-wide analysis of the rice and *Arabidopsis non-specific lipid transfer protein* (*nsLtp*) gene families and identification of wheat *nsLtp* genes by EST data mining. BMC Genomics. 2008; 9: 86 10.1186/1471-2164-9-86 18291034 PMC2277411

[37] Earley KW, Haag JR, Pontes O, Opper K, Juehne T, Song K, et al Gateway-compatible vectors for plant functional genomics and proteomics. Plant J. 2006; 45: 616–629. 16441352 10.1111/j.1365-313X.2005.02617.x

[38] Zhang XR, Henriques R, Lin SS, Niu QW, Chua NH. *Agrobacterium*-mediated transformation of *Arabidopsis thaliana* using the floral dip method. Nature Protocols. 2006; 1: 641–646. 17406292 10.1038/nprot.2006.97

[39] Mari M, Morales A, Colell A, Garcia-Ruiz C, Fernandez-Checa JC. Mitochondrial glutathione, a key survival antioxidant. Antioxid Redox Signal. 2009; 11: 2685–2700. 10.1089/ARS.2009.2695 19558212 PMC2821140

[40] Zhang B, Pasini R, Dan H, Joshi N, Zhao Y, Leustek T, et al Aberrant gene expression in the *Arabidopsis* SULTR1;2 mutants suggests a possible regulatory role for this sulfate transporter in response to sulfur nutrient status. Plant J. 2014; 77: 185–197. 10.1111/tpj.12376 24308460

[41] Gossett DR, Banks SW, Millhollon EP, Lucas MC. Antioxidant response to NaCl stress in a control and an NaCl-tolerant cotton cell line grown in the presence of paraquat, buthionine sulfoximine, and exogenous glutathione. Plant Physiol. 1996; 112: 803–809. 12226422 10.1104/pp.112.2.803 PMC158005

[42] Kader JC. Lipid-transfer proteins in plants. Ann Rev Plant Physiol Plant Mol Biol. 1996; 47: 627–654. 15012303 10.1146/annurev.arplant.47.1.627

[43] Yeats TH, Rose JK. The biochemistry and biology of extracellular plant lipid-transfer proteins (LTPs). Protein Sci. 2008; 17: 191–198. 18096636 10.1110/ps.073300108 PMC2222726

[44] Sarowar S, Kim YJ, Kim KD, Hwang BK, Ok SH, Shin JS. Overexpression of lipid transfer protein (LTP) genes enhances resistance to plant pathogens and LTP functions in long-distance systemic signaling in tobacco. Plant Cell Rep. 2009; 28: 419–427. 10.1007/s00299-008-0653-3 19089429

[45] Jung HW, Kim W, Hwang BK. Three pathogen-inducible genes encoding lipid transfer protein from pepper are differentially activated by pathogens, abiotic, and environmental stresses. Plant Cell Environ. 2003;26: 915–928. 12803619 10.1046/j.1365-3040.2003.01024.x

[46] Maldonado AM, Doerner P, Dixon RA, Lamb CJ, Cameron RK. A putative lipid transfer protein involved in systemic resistance signalling in *Arabidopsis* . Nature. 2002; 419: 399–403. 12353036 10.1038/nature00962

[47] Champigny MJ, Isaacs M, Carella P, Faubert J, Fobert PR, Cameron RK. Long distance movement of DIR1 and investigation of the role of DIR1-like during systemic acquired resistance in *Arabidopsis* . Front Plant Sci. 2013; 4: 230 10.3389/fpls.2013.00230 23847635 PMC3701462

[48] Nomura H, Komori T, Uemura S, Kanda Y, Shimotani K, Nakai K, et al Chloroplast-mediated activation of plant immune signalling in *Arabidopsis* . Nat Commun. 2012; 3: 926 10.1038/ncomms1926 22735454

[49] Zurbriggen MD, Carrillo N, Tognetti VB, Melzer M, Peisker M, Hause B, et al Chloroplast-generated reactive oxygen species play a major role in localized cell death during the non-host interaction between tobacco and *Xanthomonas campestris* pv. *vesicatoria* . Plant J. 2009; 60: 962–973. 10.1111/j.1365-313X.2009.04010.x 19719480

[50] Chen R, Sun S, Wang C, Li Y, Liang Y, An F, et al The *Arabidopsis* PARAQUAT RESISTANT2 gene encodes an S-nitrosoglutathione reductase that is a key regulator of cell death. Cell Res. 2009; 19: 1377–1387. 10.1038/cr.2009.117 19806166

[51] Bushnell WR, Perkins-Veazie P, Russo VM, Collins J, Seeland TM. Effects of deoxynivalenol on content of chloroplast pigments in barley leaf tissues. Phytopathol. 2010; 100: 33–41. 10.1094/PHYTO-100-1-0033 19968547

[52] Ansari KI, Doyle SM, Kacprzyk J, Khan MR, Walter S, Brennan JM et al Light influences how the fungal toxin deoxynivalenol affects plant cell death and defense responses. Toxins (Basel). 2014; 6: 679–692. 10.3390/toxins6020679 24561479 PMC3942759

[53] Xing F, Li Z, Sun A, Xing D. Reactive oxygen species promote chloroplast dysfunction and salicylic acid accumulation in fumonisin B1-induced cell death. FEBS Lett. 2013; 587: 2164–2172. 10.1016/j.febslet.2013.05.034 23711368

[54] Noctor G, Mhamdi A, Chaouch S, Han Y, Neukermans J, Marquez-Garcia B, et al Glutathione in plants: an integrated overview. Plant Cell Environ. 2012; 35: 454–484. 10.1111/j.1365-3040.2011.02400.x 21777251

[55] Kluger B, Bueschl C, Lemmens M, Berthiller F, Haubl G, Jaunecker G, et al Stable isotopic labelling-assisted untargeted metabolic profiling reveals novel conjugates of the mycotoxin deoxynivalenol in wheat. Anal Bioanal Chem. 2013; 405: 5031–5036. 10.1007/s00216-012-6483-8 23086087 PMC3656241

[56] Kluger B, Bueschl C, Lemmens M, Michlmayr H, Malachova A, Koutnik A, et al Biotransformation of the mycotoxin deoxynivalenol in *Fusarium* resistant and susceptible near isogenic wheat lines. PLoS One. 2015; 10(3): e0119656 10.1371/journal.pone.0119656 25775425 PMC4361057

[57] Poppenberger B, Berthiller F, Lucyshyn D, Sieberer T, Schuhmacher R, Krska R, et al Detoxification of the *Fusarium* mycotoxin deoxynivalenol by a UDP-glucosyltransferase from *Arabidopsis thaliana* . J Biol Chem. 2003; 278: 47905–47914. 12970342 10.1074/jbc.M307552200

[58] Fruhmann P, Weigl-Pollack T, Mikula H, Wiesenberger G, Adam G, Varga E, et al Methylthiodeoxynivalenol (MTD): insight into the chemistry, structure and toxicity of thia-Michael adducts of trichothecenes. Org Biomol Chem. 2014; 12: 5144–5150. 10.1039/c4ob00458b 24903010

[59] Berthiller F, Crews C, Dall'Asta C, Saeger SD, Haesaert G, Karlovsky P, et al Masked mycotoxins: a review. Mol Nutr Food Res. 2013; 57: 165–186. 10.1002/mnfr.201100764 23047235 PMC3561696

[60] Poole LB, Nelson KJ. Discovering mechanisms of signaling-mediated cysteine oxidation. Curr Opin Chem Biol. 2008; 12: 18–24. 10.1016/j.cbpa.2008.01.021 18282483 PMC2408887

[61] Mahajan N, Shi HY, Lukas TJ, Zhang M. Tumor-suppressive maspin functions as a reactive oxygen species scavenger: Importance of cysteine residues. J Biol Chem. 2013; 288: 11611–11620. 10.1074/jbc.M112.410852 23471964 PMC3630891

[62] De Coninck B, Carron D, Tavormina P, Willem L, Craik DJ, Vos C, et al Mining the genome of *Arabidopsis thaliana* as a basis for the identification of novel bioactive peptides involved in oxidative stress tolerance. J Exp Bot. 2013; 64: 5297–5307. 10.1093/jxb/ert295 24043855

[63] Singer T, Burke E. High-throughput TAIL-PCR as a tool to identify DNA flanking insertions. Methods Mol Biol. 2003; 236: 241–272. 14501069 10.1385/1-59259-413-1:241

[64] Desjardins AE, McCormick SP, Appell M. Structure-activity relationships of trichothecene toxins in an *Arabidopsis thaliana* leaf assay. J Agric Food Chem. 2007; 55: 6487–6492. 17630765 10.1021/jf0709193

