# Supplementary figures and images for: A Lipid Transfer Protein Increases the Glutathione Content and Enhances Arabidopsis Resistance to a Trichothecene Mycotoxin
Source: PLoS One. 2015 Jun 9;10(6):e0130204. doi: 10.1371/journal.pone.0130204 (PMC4461264; doi:10.1371/journal.pone.0130204)

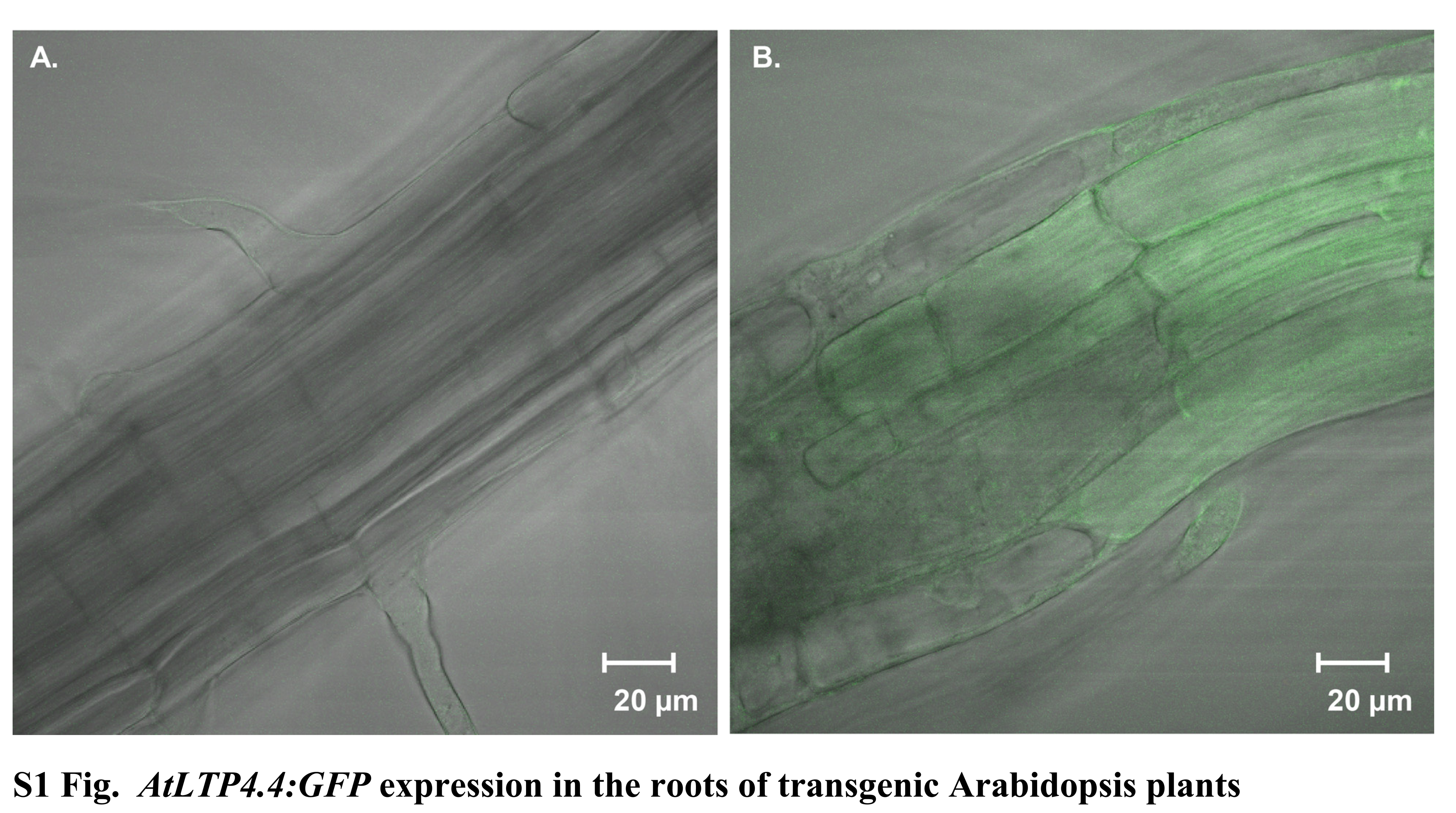

Supplement: S1 Fig — Confocal microscopy analysis of root cuttings from wild type Arabidopsis Col-0 (A) and Arabidopsis line #16 overexpressing AtLTP4.4:GFP (B). (TIF) [file pone.0130204.s001.tif]

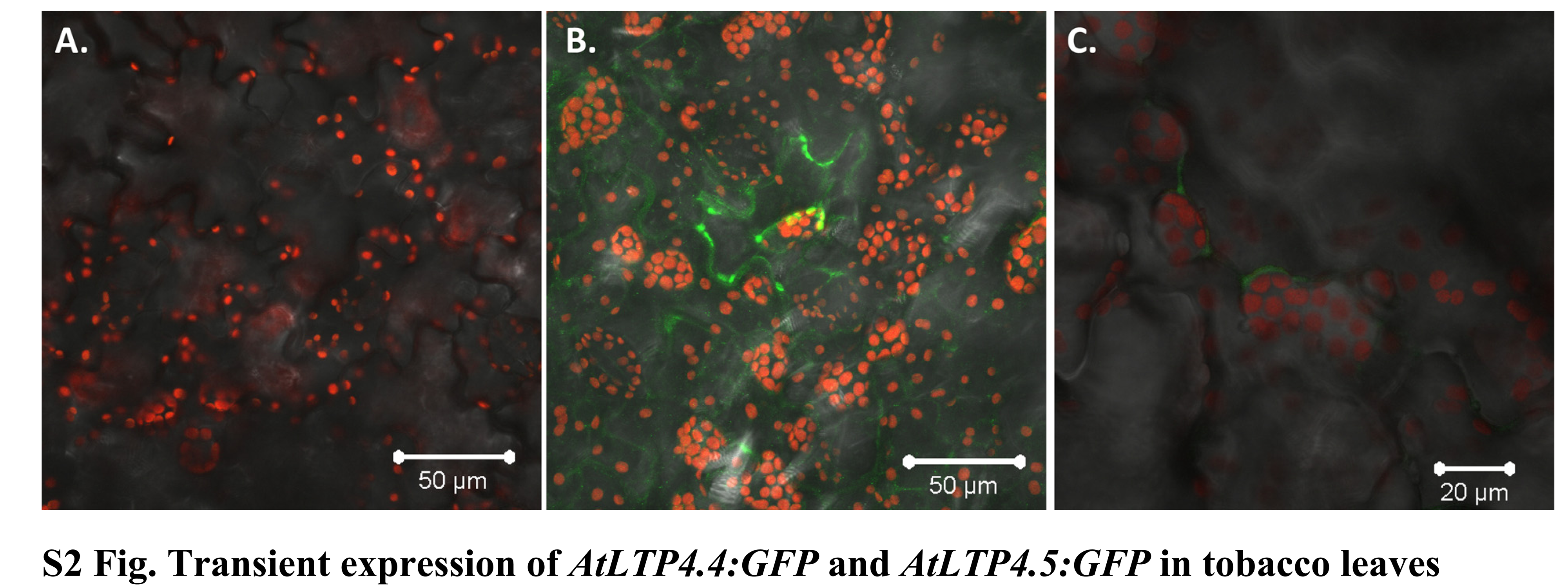

Supplement: S2 Fig — (A) Mock treated tobacco leaf with autofluorescent (red) chloroplasts. (B) Tobacco leaf infiltrated with Agrobacterium containing AtLTP4.4:GFP. (C) Tobacco leaf infiltrated with Agrobacterium containing AtLTP4.5:GFP. (TIF) [file pone.0130204.s002.tif]

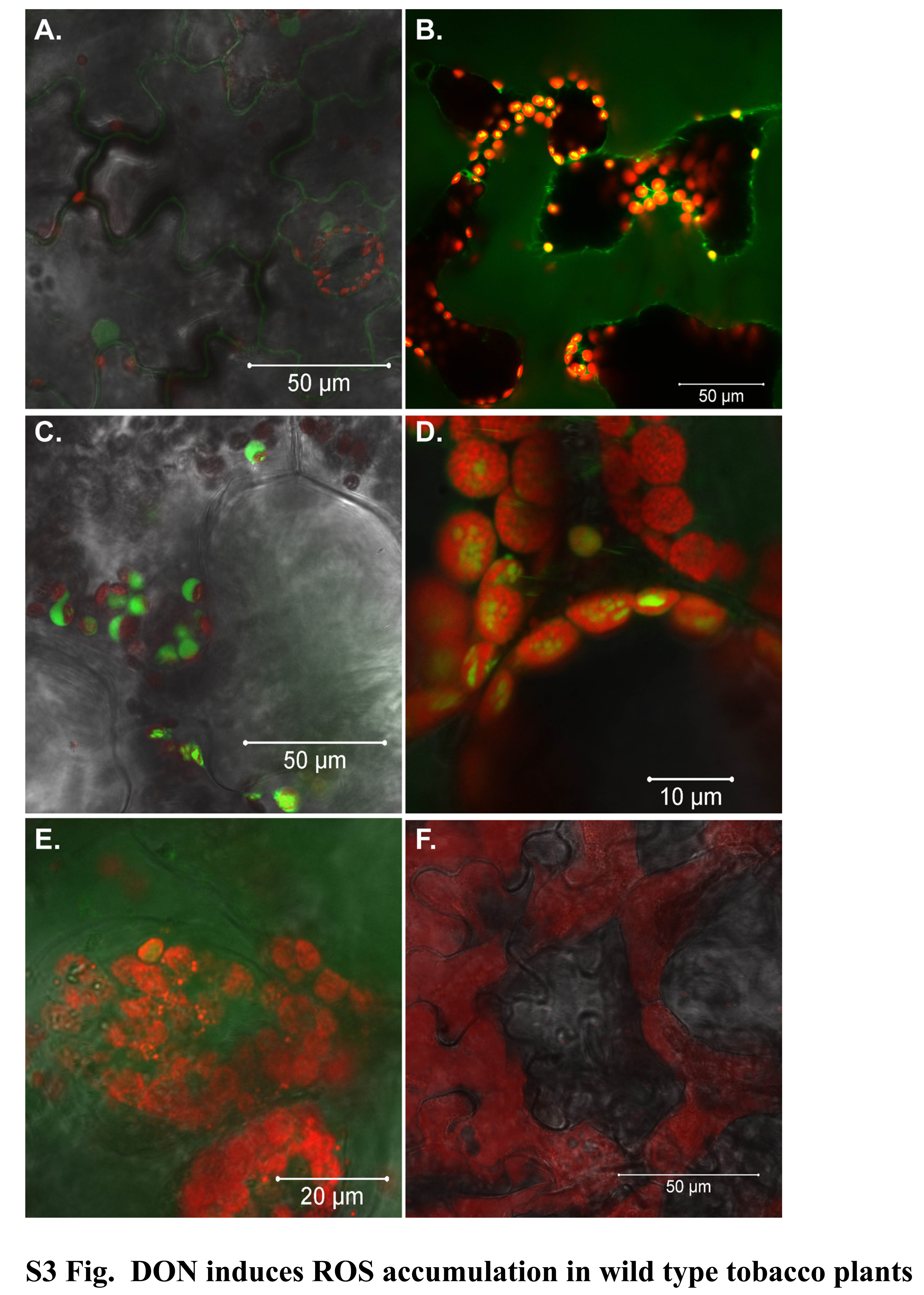

Supplement: S3 Fig — (A) Mock infiltration of tobacco leaves with buffer. (B) Infiltration of tobacco leaves with 10 μM DON for 24h, (C, D and E) infiltration of tobacco leaves with 240 μM DON for 24h. Chloroplast membrane damage is shown in (E) after 240 μM DON treatment for 24h resulting in disorganized chlorophyll autofluorescence. (F) Treatment with 600 nM paraquat for 2h. Colocalization of the DCF fluorescence (green) with chloroplasts (red) is indicated by yellow. (TIF) [file pone.0130204.s003.tif]

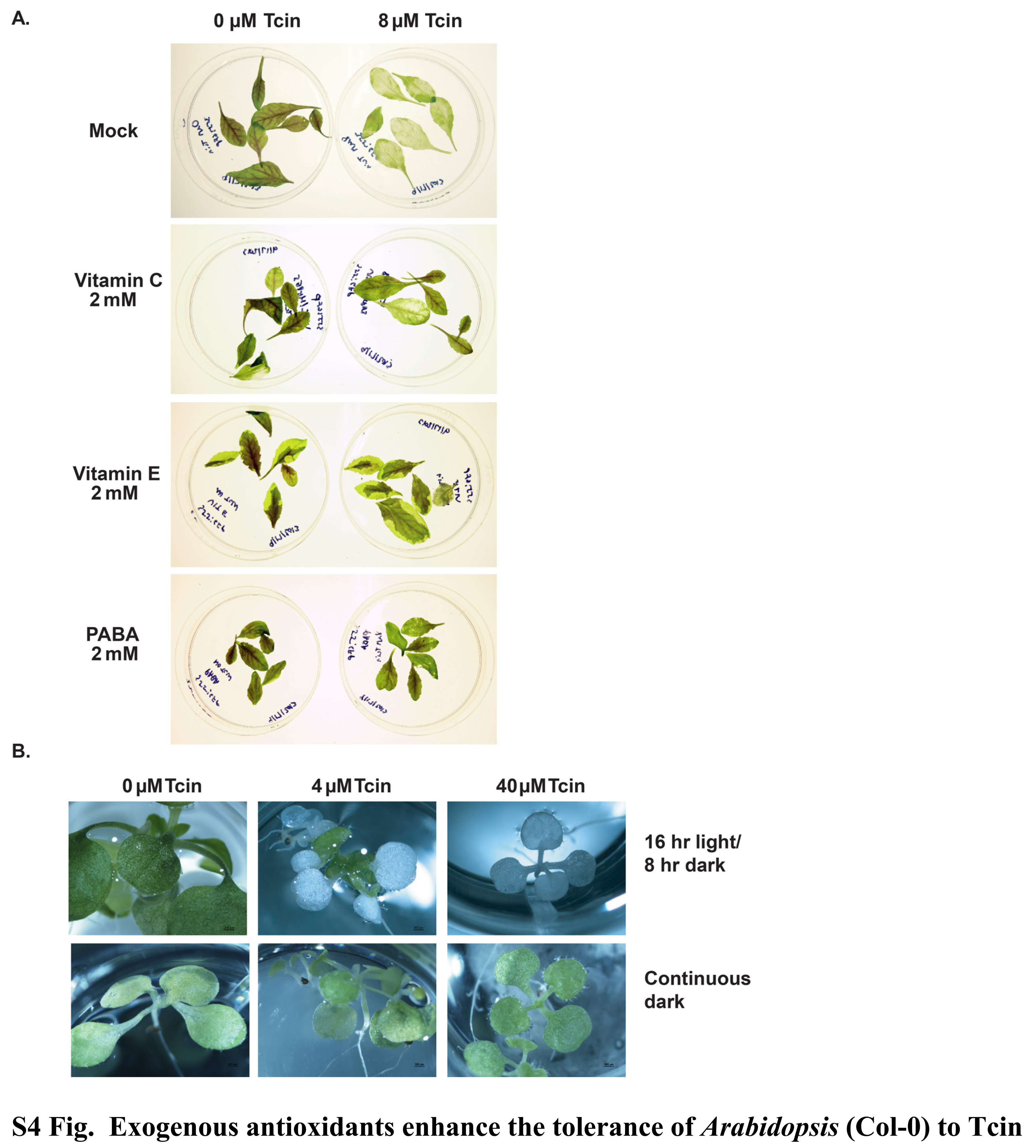

Supplement: S4 Fig — (A) Wild type Arabidopsis (Col-0) leaves were treated with 2 mM vitamin C, vitamin E, or para-amino benzoic acid (PABA) either alone or together with 8 μM Tcin for 48h and photographed. (B) Wild type Arabidopsis (Col-0) seedlings were treated with 4 μM or 40 μM Tcin and grown in 16h light/8h dark cycle or in continuous dark cycle for 6 days and photographed. (TIF) [file pone.0130204.s004.tif]

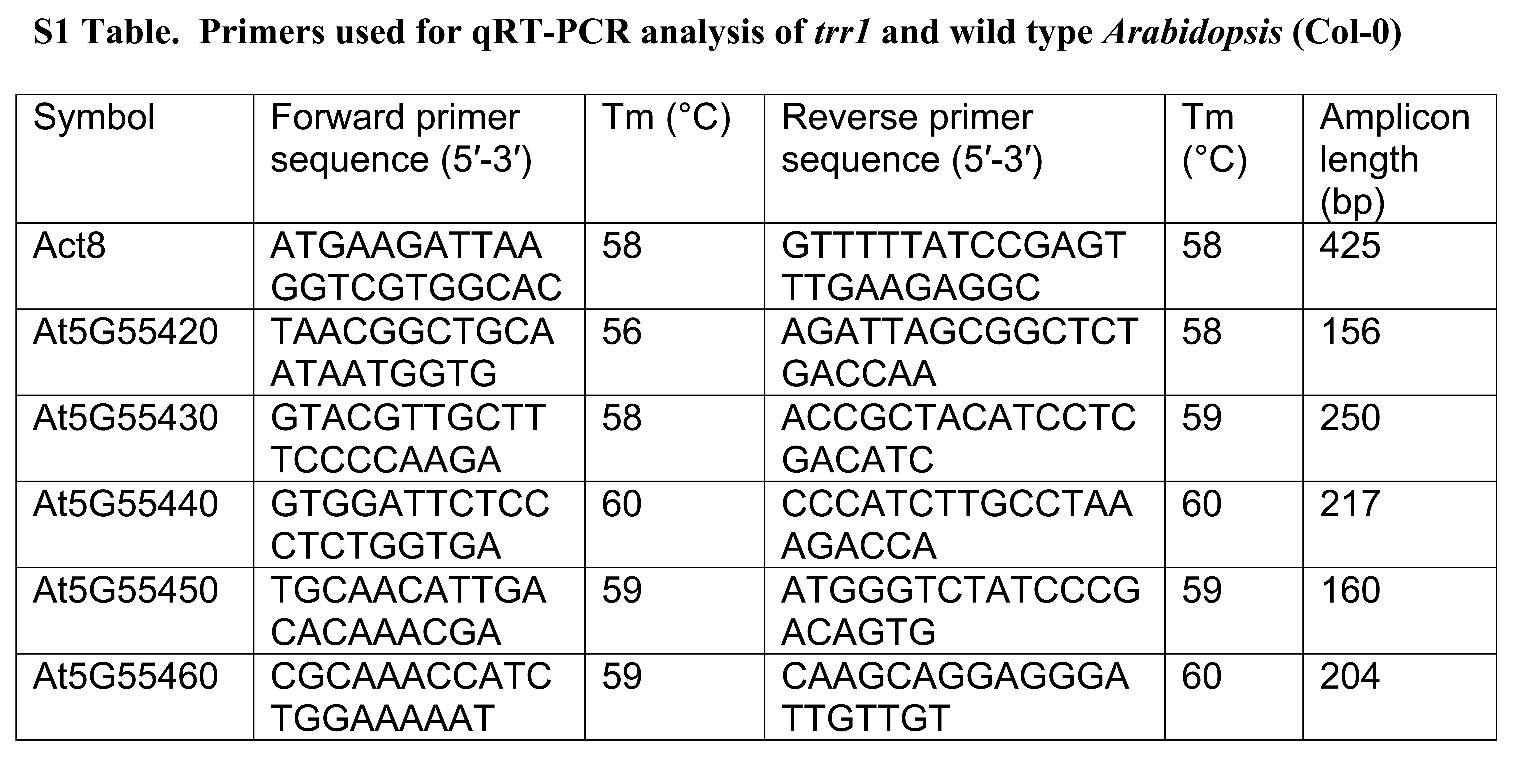

Supplement: S1 Table — Primer pair sequences used to amplify At5G55440 and upstream and downstream flanking genes and the housekeeping gene Act8 are shown. (TIF) [file pone.0130204.s005.tif]
